# Systematic enhancer mapping and functional analysis in zebrafish with optimized CRISPR interference

**DOI:** 10.1093/nar/gkaf1367

**Published:** 2025-12-18

**Authors:** Jiulin Chan, Zhichao Wu, Mingli Liu, Tianming Wang, Hanyu Liu, Ruimeng Cao, Xiaolong Li, Xinwen Li, Siyao Zhan, Jiangbo Cheng, Yicheng Xu, Mudan He, Yuanqing Feng, Qianghua Xu, Yonghua Sun, Liangbiao Chen, Peng Hu

**Affiliations:** Key Laboratory of Exploration and Utilization of Aquatic Genetic Resources, Ministry of Education, Shanghai Ocean University, Shanghai 201306, China; International Research Center for Marine Biosciences, Ministry of Science and Technology, Shanghai Ocean University, Shanghai 201306, China; Key Laboratory of Exploration and Utilization of Aquatic Genetic Resources, Ministry of Education, Shanghai Ocean University, Shanghai 201306, China; International Research Center for Marine Biosciences, Ministry of Science and Technology, Shanghai Ocean University, Shanghai 201306, China; Key Laboratory of Exploration and Utilization of Aquatic Genetic Resources, Ministry of Education, Shanghai Ocean University, Shanghai 201306, China; International Research Center for Marine Biosciences, Ministry of Science and Technology, Shanghai Ocean University, Shanghai 201306, China; Key Laboratory of Exploration and Utilization of Aquatic Genetic Resources, Ministry of Education, Shanghai Ocean University, Shanghai 201306, China; International Research Center for Marine Biosciences, Ministry of Science and Technology, Shanghai Ocean University, Shanghai 201306, China; Key Laboratory of Exploration and Utilization of Aquatic Genetic Resources, Ministry of Education, Shanghai Ocean University, Shanghai 201306, China; International Research Center for Marine Biosciences, Ministry of Science and Technology, Shanghai Ocean University, Shanghai 201306, China; Key Laboratory of Exploration and Utilization of Aquatic Genetic Resources, Ministry of Education, Shanghai Ocean University, Shanghai 201306, China; International Research Center for Marine Biosciences, Ministry of Science and Technology, Shanghai Ocean University, Shanghai 201306, China; The State Key Laboratory of Grassland Agro-ecosystems, College of Pastoral Agriculture Science and Technology, Lanzhou University, Lanzhou, Gansu 730020, China; Key Laboratory of Exploration and Utilization of Aquatic Genetic Resources, Ministry of Education, Shanghai Ocean University, Shanghai 201306, China; International Research Center for Marine Biosciences, Ministry of Science and Technology, Shanghai Ocean University, Shanghai 201306, China; Key Laboratory of Exploration and Utilization of Aquatic Genetic Resources, Ministry of Education, Shanghai Ocean University, Shanghai 201306, China; International Research Center for Marine Biosciences, Ministry of Science and Technology, Shanghai Ocean University, Shanghai 201306, China; The State Key Laboratory of Grassland Agro-ecosystems, College of Pastoral Agriculture Science and Technology, Lanzhou University, Lanzhou, Gansu 730020, China; Key Laboratory of Exploration and Utilization of Aquatic Genetic Resources, Ministry of Education, Shanghai Ocean University, Shanghai 201306, China; International Research Center for Marine Biosciences, Ministry of Science and Technology, Shanghai Ocean University, Shanghai 201306, China; State Key Laboratory of Freshwater Ecology and Biotechnology, Key Laboratory of Breeding Biotechnology and Sustainable Aquaculture, Institute of Hydrobiology, Innovation Academy for Seed Design, Chinese Academy of Sciences, Wuhan 430072, China; Department of Genetics, University of Pennsylvania, Philadelphia, PA 19104, United States; Key Laboratory of Exploration and Utilization of Aquatic Genetic Resources, Ministry of Education, Shanghai Ocean University, Shanghai 201306, China; International Research Center for Marine Biosciences, Ministry of Science and Technology, Shanghai Ocean University, Shanghai 201306, China; State Key Laboratory of Freshwater Ecology and Biotechnology, Key Laboratory of Breeding Biotechnology and Sustainable Aquaculture, Institute of Hydrobiology, Innovation Academy for Seed Design, Chinese Academy of Sciences, Wuhan 430072, China; Key Laboratory of Exploration and Utilization of Aquatic Genetic Resources, Ministry of Education, Shanghai Ocean University, Shanghai 201306, China; International Research Center for Marine Biosciences, Ministry of Science and Technology, Shanghai Ocean University, Shanghai 201306, China; Key Laboratory of Exploration and Utilization of Aquatic Genetic Resources, Ministry of Education, Shanghai Ocean University, Shanghai 201306, China; International Research Center for Marine Biosciences, Ministry of Science and Technology, Shanghai Ocean University, Shanghai 201306, China; Marine Biomedical Science and Technology Innovation Platform of Lin-gang Special Area, Shanghai 201306, China

## Abstract

Noncoding *cis-*regulatory elements, particularly enhancers, are crucial for controlling gene expression. However, the *in vivo* use of Clustered Regularly Interspaced Short Palindromic Repeats (CRISPR) interference (CRISPRi) to study enhancer function has been limited in zebrafish, which is widely used in early development and human disease research. Here, we optimized the CRISPRi system in zebrafish to achieve efficient suppression of *tyr* expression by fine-tuning component concentrations. Applying this optimized system, we functionally annotated distal enhancers of globin genes. Using Hi-C and histone modification assays, we systematically mapped 434 enhancer–promoter (EP) interactions across the genome. Among these EP loops, CRISPRi perturbation identified previously unreported enhancers with regulatory strengths surpassing known elements, demonstrated by disrupted phenotypes in fin and blood cell development. Additionally, several unreported EP loops were validated, underscoring the robustness of our integrated approach. This study not only provides an optimized CRISPRi system for zebrafish but also introduces a powerful platform that integrates computational and experimental strategies for advancing *cis-*regulatory element annotation in vertebrate gene regulation.

## Graphical Abstract

**Graphical Abstract:**
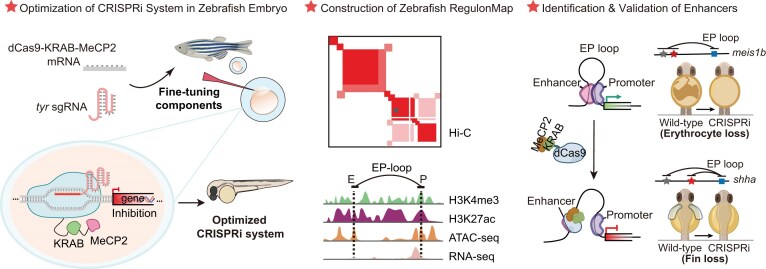

## Introduction

Enhancers are critical gene-regulatory elements that control precise functional gene expression by engaging in physical interactions with the promoter regions of their target genes, often through long-range chromosomal contacts [1, 2]. These interactions are essential for orchestrating complex processes such as cell differentiation during development. Disruptions of enhancer sequences or their physical interactions with promoters can severely impact normal development and lead to disease [3, 4]. For example, the locus control region (LCR) interacts with the β-globin promoter to regulate blood development [5, 6], while the zone of polarizing activity regulatory sequence (ZRS) controls sonic hedgehog (*shh*) expression for limb development [7]. Additionally, sequence variation in enhancers has been shown to influence developmental phenotypes, contributing to evolutionary adaptations [8].

Enhancers are often located hundreds of kilobases (kb) away from the genes they regulate, bypassing proximal genes to selectively interact with their targets [9, 10]. Despite their significance, functionally annotating enhancers remains a central challenge in genome biology due to two main hurdles: (i) the lack of high-resolution maps identifying putative enhancers across the genome, and (ii) the need for methods to functionally validate the regulatory relationships between distal enhancers and proximal promoters of functional genes.

For the first challenge, advances in genomic assays such as Assay for Transposase-Accessible Chromatin with high-throughput sequencing (ATAC-seq, to identify open chromatin regions) [11], Chromatin Immunoprecipitation Sequencing (ChIP-seq) and Cleavage Under Targets and Tagmentation (CUT&Tag) (to profile protein–DNA interactions) [12], and High-throughput Chromosome Conformation Capture (Hi-C, to study 3D chromatin architecture) [13] have provided powerful tools to map chromatin signatures and enhancer–promoter (EP) interactions in animal systems. These tools enable genome-wide mapping of regulatory interactions and have significantly advanced our understanding of enhancer biology [14, 15].

For the second challenge, Clustered Regularly Interspaced Short Palindromic Repeats (CRISPR) technology offers a groundbreaking solution. Originally discovered as a microbial immune system, CRISPR has revolutionized molecular biology by enabling precise and programmable targeting of nucleic acid [16, 17]. Beyond genome editing, CRISPR-mediated activation (CRISPRa) and interference (CRISPRi) adapt the system for gene regulation without altering DNA sequences [18]. CRISPRi employs a catalytically inactive Cas9 protein (dCas9) fused with transcriptional repressors such as krüppel-associated box (KRAB) [19], Lysine-specific demethylase 1 (LSD1) [20], zinc finger imprinted 3 (Zim3) [19], or methyl-CpG binding protein 2 (MeCP2) [21], or with DNA methyltransferase 3 alpha (Dnmt3A) and DNA methyltransferase 3 like (Dnmt3L) [22]. These systems allow stable repression, making them ideal for studying noncoding regulatory elements, including distal enhancers.

Despite these advances, CRISPRi applications for targeting enhancers remain underexplored, particularly *in vivo*. Most studies focus on proximal promoter regions, with enhancer functions largely characterized *in vitro* [23, 24]. The *in vivo* application of CRISPRi to study enhancers during development is hampered by challenges such as optimizing single-guide RNA (sgRNA) design, determining the ideal concentration ratio of dCas9 to sgRNA [25], balancing efficacy with toxicity [26], ensuring long-lasting transcriptional repression, and minimizing off-target effects [27]. These factors have yet to be comprehensively investigated in an organismal context.

Zebrafish provide an exceptional model for addressing these gaps. Their *ex vivo* embryo development, transparent early-stage phenotypes, and amenability to high-throughput genetic manipulation make them an ideal system for studying gene regulation. Zebrafish are widely used for investigating blood, heart, and neural development, as well as related diseases, and offer potential for drug screening and therapeutic discovery [28, 29]. However, systematic application of CRISPRi in zebrafish to investigate enhancers remains unreported.

In this study, we present a systematically optimized CRISPRi system for zebrafish, tailored for enhancer function analysis. By integrating computational and experimental approaches, we mapped EP interactions genome-wide using multi-omic datasets, including Hi-C, ATAC-seq, and histone modification profiling. Our approach enabled functional validation of both known and novel enhancers in zebrafish embryos. This work not only establishes an effective platform for studying enhancer functions *in vivo* but also provides a comprehensive resource for elucidating gene regulation in vertebrate development, evolution, and diseases.

## Materials and methods

### Animal maintenance

Wild-type zebrafish embryos were obtained by breeding male and female AB strain fish. All zebrafish experiments were conducted according to the guidelines of the Committee on Laboratory Animal Care and Use of Shanghai Ocean University.

### sgRNAs design and synthesis

Based on the idea that sgRNAs targeting transcriptionally active and accessible chromatin are more effective in CRISPRi-mediated repression [30], we refined our sgRNA design by integrating chromatin accessibility and histone modification data in our study. Specifically, we aligned sgRNAs with H3K4me3 (marking promoter regions), H3K27ac (marking active transcription) peaks, and open chromatin as indicated by ATAC-seq signals.

sgRNAs were designed to minimize off-targets using the publicly available tool CHOPCHOP [31] (https://chopchop.cbu.uib.no/) with the setting “20bp-NGG-SpCas9.” The top three sgRNAs were picked based on the following criteria: (i) target sites with no predicted mismatches, (ii) high-efficiency scores (>60), and (iii) minimal off-target potential in other regions of the zebrafish genome, as indicated by the lowest MM0, MM1, MM2, and MM3 values. Linearized DNA templates of sgRNAs used for embryo injection were synthesized by Sangon Biotech (Shanghai) Co., Ltd. with a T7 promoter sequence at the base. Subsequently, sgRNA was produced utilizing the MAXIscript T7 transcription kit (ThermoFisher, AM1314), following the manufacturer’s protocol with the 1 μg linearized DNA template. The sgRNA was purified and stored at −80°C for future applications. All sgRNA sequences are listed in Supplementary Table S1.

### dCas9 mRNA synthesis

For the CRISPRi assays, the dCas9-KRAB-MeCP2 template plasmid was obtained from Addgene (Cat#122205) and amplified by polymerase chain reaction (PCR) using the following primers: forward primer: 5′-ctccacctaagaagaagagaaaggtgggaagcatggacaagaagtacagcatcg-3′ and reverse primer: 5′-ctggtttagtggtaaccagatccgcggtcatgagactctctcagtcacgg-3′. The PCR fragment was inserted into the pZcas9 plasmid [32] between the AgeI and NcoI sites using Gibson assembly (NEB, Cat#E2621). The plasmid was then linearized with XbaI (NEB, Cat#R0145S) to serve as a transcription template. In addition, the KRAB-MeCP2 domain-deleted dCas9 alone plasmid was generated by PCR amplification from the dCas9-KRAB-MeCP2 plasmid. Two fragments were amplified by using the following primers: dCas9-F1 5′-gtgggagcaacggcagcagcggatcctgaccgcggatctggttacc-3′, dCas9-R1 5′-cctcgaacagctggttgtaggtc-3′; dCas9-F2 5′-gacaagctgttcatccagctggtg-3′, dCas9-R2 5′-gtggtaaccagatccgcggtcaggatccgctgctgccgttg-3′, respectively. And then, the two fragments were circularized using Gibson assembly (NEB, Cat#E2621), the reconstructed plasmid was confirmed by Sanger sequencing.

The dCas9-KRAB-MeCP2 and dCas9 alone mRNA were transcribed *in vitro* using the mMESSAGE mMACHINE^™^ T3 Transcription Kit (Thermo Fisher Scientific, Cat#AM1348) in a reaction system containing nucleoside triphosphates (NTPs). After transcription, the reaction was treated with DNase I (Thermo Fisher Scientific, Cat#EN0521) to remove any remaining DNA, followed by LiCl precipitation to purify the mRNA. The concentration of the purified dCas9 mRNA was measured using a NanoDrop spectrophotometer and stored at −80°C.

### Collection of zebrafish embryos and micro-injection

Zebrafish embryos were collected and aligned in a 1% agarose-coated dish. Microinjections were performed with a finely pulled glass capillary needle under a dissecting microscope. To knockout the *tyr* gene, ~1 nl of sgTyr and Cas9 mRNA mixtures were injected into the blastodisc of one-cell stage embryos. These embryos were then cultured at 28°C and subsequently evaluated for phenotypic changes, genotyping, and mutation efficiency. To determine the optimal sgRNA-to-dCas9 ratio for *in vivo* CRISPRi activity in zebrafish, ~1 nl of sgRNA/dCas9 mRNA mixtures with different ratios (1:1 = 25:25 ng/μl; 1:4 = 25:100 ng/μl; 1:8 = 25:200 ng/μl) were injected into the blastodisc of one-cell stage embryos. After establishing the optimal ratio, we prepared additional sgRNA and dCas9 mRNA mixtures at various final concentrations (12.5:50 ng/μl; 25:100 ng/μl; 50:200 ng/μl; 100:400 ng/μl) and injected them into one-cell stage embryos. Following injection, the embryos were cultured at 28°C. For each CRISPRi experiment, three to five independent microinjections were performed to collect embryos for subsequent analysis of survival rate estimation, developmental phenotypes, quantitative RT-PCR (qRT-PCR).

### Analysis of *tyr* knockout zebrafish

For assessing mutation phenotypes of melanin deposition, 36 and 48 h post-injection (hpi) embryos edited by targeting *tyr*, and wild-type embryos were evaluated under the same microscopic setup using an Axio Zoom.V16 stereo microscope (Zeiss) equipped with an Axiocam 705 color camera. To verify *tyr* knockout at the DNA level, we performed targeted Sanger sequencing of the genomic region flanking the sgRNA-binding sites. The corresponding genotyping primers were listed in Supplementary Table S1. To estimate the proportion of edited cells in edited embryos, we followed the approach as previously described [33]. Specifically, genomic DNA was extracted from individual edited embryos, and the *tyr* locus was amplified by PCR using the corresponding genotyping primers. A T7 endonuclease 1 (T7E1) endonuclease assay was then performed to detect indels. The mutation efficiency was assessed by calculating the ratio of the intensity of the cleaved bands to the total intensity of all bands after enzyme digestion.

### *O*-Dianisidine staining and image acquisition of zebrafish embryos

*O*-Dianisidine staining for hemoglobin production and erythrocyte formation was conducted as previously described [34]. In brief, zebrafish embryos were dechorionated with pronase and incubated in *o*-dianisidine solution (0.62 mg/ml *o*-dianisidine, 0.65% (v/v) hydrogen peroxide (H_2_O_2_,30% stock), and 43% (v/v) ethanol in 10 mmol/l sodium acetate) in the dark for 15 min at room temperature. The reaction was stopped by adding Phosphate Buffered Saline with Tween-20 (PBST), followed by three rinses with PBS. After staining, embryos were bleached with a solution containing 0.8% (w/v) potassium hydroxide (KOH), 0.9% (v/v) H_2_O_2_, and 0.1% (v/v) Tween-20, then stored in 50% (v/v) glycerol.

Stained embryos were mounted on glass-bottom dishes with the yolk sac facing upward. Z-stacked, bright-field images were captured on an Axio Zoom.V16 stereo microscope (Zeiss) equipped with an Axiocam 705 color camera. All images were acquired at 3.2 × zoom, 2464 × 2056 pixels, 42 bit RGB, with identical light settings across samples. Uncompressed CZI (Carl Zeiss Image) format was used to preserve image fidelity. Calibration was performed using a stage micrometer to record pixel-to-micrometer conversion. Images with clear and sharp stain area were included in the analysis, to minimize staining and imaging artefact.

### Phenotypic assessments

For hemoglobin production and erythrocyte formation analysis, embryos injected with CRISPRi components targeting the LCR of globin genes, and the myeloid ecotropic viral integration site 1b (*meis1b*) enhancers (E9 and the novel enhancer Enovel) were assessed at 48 hpi. Workflow for quantifying hemoglobin-positive signal was performed as follow: Raw images (.czi format) of *o*-dianisidine staining were imported into Fiji [35] (ImageJ 2.14.0/1.54f; Java 1.8.0_322 [64-bit]). To ensure reproducibility, all processing steps were executed in batches. A representative image was used to manually define the optimal thresholding parameters in the Color Threshold tool. These settings specifically isolated the brown peroxidase reaction product (*o*-dianisidine–hemoglobin complex). The threshold values (for instance: *B* = 105, *G* = 150, *R* = 190) were saved and uniformly applied to all embryos within the batch process to minimize inter-sample variability. Two binary masks were created for hemoglobin positive area and yolk sac area. Pixels within each mask were counted and hemoglobin index for each embryo was calculated as: “Hemoglobin-positive area (pixels)” divided by “Yolk sac area (pixels).” Embryos with incomplete yolk sacs or aberrant morphology (e.g. coagulation and tissue damage) were excluded.

For assessing developmental phenotypes of pectoral fins, 72 hpi embryos modulated by targeting the *shh* ZRS and its shadow enhancer (sZRS), novel element, and the ZRS + novel element region identified in this study, were evaluated under the same microscopic setup.

### Survival rate estimation

Survival rates were measured to evaluate the viability of embryos injected with the CRISPRi components (sgRNA/dCas9 mRNA mixtures, Single-component dCas9 or sgRNA) with varying final concentration. In each group, >100 (106–322) embryos from five independent micro-injections were selected, and survivors was counted. The survival rate was calculated as the ratio of surviving embryos to the total number of embryos. Each experiment was repeated five times, and the mean survival rate was determined.

### RNA isolation and qRT-PCR analysis

Total RNA was isolated from zebrafish embryos at 48 or 72 hpi using Trizol reagent (Thermo Fisher Scientific, Cat#15596018CN) following the manufacturer’s protocol. Each experiment was performed in three independent biological replicates, with more than six samples collected per replicate. For each sample, 3–5 injected embryos were pooled and homogenized in 500 μl of Trizol reagent. After incubation at room temperature, 100 μl of chloroform was added, and the mixture was vortexed for 15 s, then centrifuged to separate the phases. The upper aqueous phase, containing RNA, was carefully transferred to a new tube, and an equal volume of isopropanol was added to precipitate the RNA. The RNA pellet was washed with 75% (v/v) ethanol, resuspended in 20 μl of RNase-free water, and its concentration and quality were measured using a NanoDrop spectrophotometer.

For qRT-PCR, total RNA of each sample was reverse-transcribed using ABScript Neo RT Master Mix (Abclonal, Cat#RK20433) according to the manufacturer’s instructions. qRT-PCR was performed in duplicate using SsoFast Eva Green Supermix (Bio-Rad, Cat#1725201) on a CFX Opus 96 Real-Time PCR System (Bio-Rad). The PCR amplification protocol consisted of an initial step at 95°C for 30 s, followed by 40 cycles of 95°C for 10 s and 58°C for 30 s. Primer sequences are provided in Supplementary Table S1. Analysis of qRT-PCR was determined using the 2^−ΔΔCt^ method [36] with *β-actin* as the reference gene.

### Hi-C library preparation and data analysis

Hi-C library preparation was conducted with minor modifications as previously described [37]. Wild-type zebrafish embryos at 48 h post-fertilization (hpf) were used, with an input of three to five million nuclei. Below is a detailed description of the procedure:

#### Nuclei extraction and cell cross-linking

Approximately 400 embryos were pooled and snap-frozen in liquid nitrogen. Embryos were then homogenized in 2 ml of freshly prepared homogenization buffer [30 mM calcium chloride, 18 mM magnesium acetate, 66 mM Tris pH 8.0, 1 mM β-mercaptoethanol, 0.1 mM phenylmethylsulfonyl fluoride (PMSF), 320 mM sucrose, 0.1 mM ethylenediaminetetraacetic acid (EDTA), 0.1% (v/v) Nonidet *P*-40, and 1× protease inhibitors (Roche, Cat#5892970001)] using a Dounce Homogenizer on ice. Large tissue debris was removed by centrifugation at 100 × *g* for 1 min at 4°C, followed by 750 × *g* for 5 min to pellet the nuclei. The pellet was washed with 0.04% phosphate-buffered saline–bovine serum albumin (PBS–BSA) buffer, then fixed in 1% (w/v) paraformaldehyde (diluted in 0.04% PBS–BSA) for 10 min at room temperature. Fixation was stopped by adding glycine to a final concentration of 0.2 M and incubating for 5 min. The nuclei were washed with 1× PBS and prepared for chromatin digestion.

#### Chromatin digestion

Nuclei were resuspended in 1 ml of Hi-C lysis buffer [10 mM Tris (hydroxymethyl) aminomethane hydrochloride (Tris-HCl, pH 8.0), 10 mM Sodium chloride, 0.2% (v/v) Igepal, and 1× protease inhibitors (Roche, Cat#5892970001)] and incubated on ice for 20 min, vortexing every 5 min. After centrifugation, the pellet was resuspended in 250 μl of 0.5% (v/v) sodium dodecyl sulfate (SDS, 10% stock) and incubated at 62°C for 7 min. After adding 787.5 μl of water and 62.5 μl of 20% (v/v) Triton X-100, samples were incubated at 37°C for 5 min. Sample was digested by adding 500 μl of 1× 0.1% (v/v) Triton X-100 containing rCutSmart™ Buffer (NEB, Cat#B6004V) and 400 units of HaeIII and AluI (NEB, Cat#R0108S and Cat#R0137) and incubating overnight at 37°C.

#### A-tailing, proximity ligation, and linker removal

Digestion mixture was processed with 400 μl of Klenow (3′-5′ exo-) solution [40 μl of NEBuffer™ 2 (NEB, Cat#B7002S), 8 μl of 10 mM dATP (NEB, Cat#N0440S), 40 μl of 10% (v/v) Triton X-100, 308 μl of H_2_O, and 4 μl of Klenow (NEB, Cat#M0212S)] at 37°C for 1 h. Nuclei were pelleted and washed with 1× PBS and 1× T4 DNA Ligase Reaction Buffer (NEB, Cat#B0202S). Proximity ligation was performed in 1200 μl of ligation solution [946 μl of H_2_O, 120 μl of T4 DNA ligase buffer, 120 μl of 10% (v/v) Triton X-100, 8 μl of T4 DNA ligase (NEB, Cat#M0202S), 6 μl of 10% (v/v) BSA, and 4 μl of bridge linker] and incubated overnight at 4°C. To remove open DNA and linkers, 700 μl of exonuclease mix [70 μl of 10× Lambda Exonuclease Reaction Buffe (NEB, Cat#B0262), 3 μl of lambda exonuclease (NEB, Cat#M0262L), 3 μl of exonuclease I (NEB, Cat#M0293S), 624 μl of H_2_O, and 3.5 μl of 10% (v/v) Triton X-100] was added and incubated at 37°C for 1 h.

#### Reverse crosslinking and DNA purification, biotin capture, and sequencing

Nuclei were washed with 1× PBS [containing 0.1% (v/v) Triton X-100] and resuspended in 500 μl of Protease Lysis buffer [10 mM Tris-HCl pH 8.0, 100 mM sodium chloride, 25 mM ethylenediaminetetraacetic acid, 0.5% SDS (10% stock), and 0.2 mg/ml protease K (20 mg/ml, Invitrogen, Cat#25530049)] for 10 h at 65°C. The supernatant was collected, and RNA was precipitated with ethanol, centrifuged, and dried. DNA was dissolved in 140 μl of Tris buffer (10 mM Tris-HCl pH 8.0). Biotinylated DNA was sheared to 300–500 bp using Covaris M220 (130 μl volume, 20 μg DNA, 300 bp target, 50 power, 20% duty, 200 cycles, 65 s). DNA was transferred to a new tube, rinsed with Tris-ethylenediaminetetraacetic acid buffer, bringing the final volume to 200 μl. Biotin-labeled DNA was bound to 15 μl of Dynabeads MyOne Streptavidin T1 beads (Life Technologies, Cat#65602) per the manufacturer’s instructions. Beads were washed with Tween-washing buffer and resuspended in binding buffer, mixed with DNA, and incubated for 30 min at room temperature. After washing, beads were resuspended in 50 μl of 5 mM Tris (pH 8.0) for TruSeq library preparation. DNA end repair and adaptor ligation were performed with the NEBNext^®^ Ultra^™^ II End Repair/dA-Tailing Module (NEB, Cat#E7546S). End repair was performed at 20°C for 30 min and at 65°C for 30 min, followed by adaptor ligation at 20°C for 15 min. USER^®^ Enzyme (3 μl, NEB, Cat#M5505L) was added, and the reaction was incubated at 37°C for 15 min. Beads were washed and resuspended in 20 μl of 5 mM Tris (pH 8.0). The final library was prepared with KAPA HiFi HotStart ReadyMix (Roche, Cat#07958927001) using a PCR protocol: 95°C for 3 min, 5 cycles of 98°C for 10 s, 65°C for 30 s, 72°C for 30 s, and a final extension at 72°C for 1 min. The library was purified with SPIRV beads and sequenced on the NovaSeq X Plus platform in PE150 mode by Haplox Biotechnology Co., Ltd.

#### Data analyses

Paired-end Hi-C fastq files were merged using FLASH v1.2.11[38] with parameters “-m 25 -M 150 -t 8 -p 33.” Linker trimming was performed using the python scripts as described in https://github.com/fengyq/my_python_scrpts/blob/main/split_linker_mergedSE.py and https://github.com/fengyq/my_python_scrpts/blob/main/split_linker_PEv2.py with the default settings. Low-quality reads were filtered out using fastp v0.23.4 [39], with parameters “–disable_adapter_trimming –disable_quality_filtering –length_required 20.” The resulting high-quality reads were aligned to the zebrafish genome assembly GRCz11 (GCF_000002035.6) using BWA [40] with default parameters. Ligation events were detected, sorted, and PCR duplicates were removed using the pair tools package (https://github.com/mirnylab/pairtools). The multiresolution hic file was generated using Juicer Tools v1.22.01 pre [41], producing KR-normalized matrices at 5, 10, and 25-kb resolutions. Chromatin loops were subsequently identified with Juicer Tools hiccups [41] using parameters “-r 25 000/10 000/5000 -k KR –ignore-sparsity”. Chromatin loop signals were visualized with Juicebox v1.11.08 [42].

#### EP-loops identification

To identify EP loops, we utilized narrowPeak files from ChIP-seq (H3K4me3 and H3K27ac) and ATAC-seq datasets. Peaks with log*P* > 5 were selected and overlapping signals were identified using bedtools v2.27.0 [43]. Promoters were defined as regions exhibiting three overlapping signals (H3K4me3, H3K27ac, and ATAC-seq), while enhancers were identified as regions with overlapping ATAC-seq and H3K27ac signals but lacking H3K4me3. EP-loops were then defined as chromatin loops connecting these enhancers and promoters. The EP-loop signals were visualized with Juicebox and the Integrative Genomics Viewer (IGV) [44].

### Public datasets used in this study and data processing

The ATAC-seq and RNA-seq datasets for 48 hpf zebrafish embryos [45] used in this study are available in the GEO database (accession code: GSM4724550 and GSM4724538). ChIP-seq datasets for H3K4me3 and H3K27ac in 48 hpf zebrafish embryos [46] were obtained from the DANIO-CODE database (https://danio-code.zfin.org/dataExport/). The bed file for ultra-conserved non-coding elements (UCNEs) in the zebrafish GRCz7 genome (UCNEs_danRer7.bed) was sourced from the UCNE database (http://ccg.vital-it.ch/UCNEbase/), and converted to the GRCz11 assembly using the UCSC LiftOver tool (https://genome.ucsc.edu/cgi-bin/hgLiftOver).

Quality control on sequencing reads from these datasets was performed with fastp v0.23.4 [39]. Paired reads were aligned to the zebrafish reference genome (GRCz11, GCF_000002035.6) using Bowtie2 v2.3.4.2 [47] with parameters “–no-mixed –no-discordant -X 700.” PCR duplicate reads were removed with Picard MarkDuplicates [48]. Signal peaks were called using MACS2 v2.1.0 [49] with parameters “–bdg -q 0.05 –nomodel.” Bigwig files were generated using the bamCoverage function in deeptools v2.0.0 [50] with options “–normalizeUsing RPKM -p 20 –binSize 1 –filterRNAstrand forward,” and signal tracks were visualized using IGV.

### Construction of interactive web portal

The interactive web portal was developed using the R Shiny framework [51], incorporating the JBrowseR package [52] for interactive genomic visualization. Genomic datasets, including fasta, gff, narrowPeak, bw, and bed files, were integrated into JBrowseR to enable detailed exploration of the genomic data. The data.table package was used to efficiently process and manage bed files, allowing rapid manipulation and visualization of large-scale data. A help interface is provided within the application to give users an overview of loaded files, explaining their content and relevance to the project.

### Statistical analysis

All data are presented as the mean or the mean ± standard error of the mean (SEM) from at least three independent experiments. One-way ANOVA and post hoc Bonferroni correction were used to evaluate differences in gene expression levels among groups. Chi-square tests were used to analyze the categorical data, including the phenotypes of embryo survival post-injection, fin-ray development, and erythrocyte formation. When performing pairwise comparisons, the significance level *P* is adjusted using the post hoc Bonferroni correction according to the formula *P *= 2*α*/k*(*k* − 1), where *k* is the number of groups being compared. Here, *α* is set at 0.05. This correction helps control the family-wise error rate across multiple comparisons. Sample sizes were not predetermined by power analysis but were based on standards in the field. Full statistical details, including sample size (*n*), means, and statistical significance (adjust *P*), are provided in the corresponding figure legends. All statistical comparisons were conducted using SPSS version 25.0 (IBM Corp., Armonk, NY, USA).

## Results

### Efficient CRISPRi-mediated repression of the *tyr* gene in zebrafish embryos

**Figure 1.**
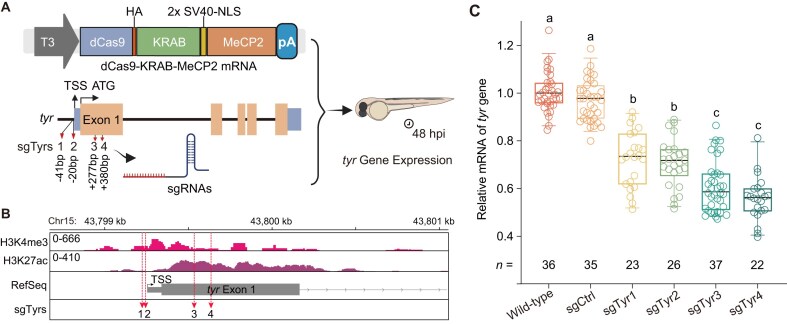
CRISPRi-mediated repression of *tyr* transcription in zebrafish embryos. (**A**) Schematic of the CRISPRi strategy used to repress *tyr* expression. A nuclease-inactive Cas9 (dCas9) was fused to dual repressor domains—Krüppel-associated box (KRAB) and methyl-CpG-binding protein 2 (MeCP2)—and co-injected as *in vitro* transcribed mRNA with sgRNAs targeting the proximal *tyr* promoter (sgTyr1-4; −50 bp to + 400 bp relative to the transcription start site, TSS). HA, human influenza hemagglutinin epitope tag; SV40-NLS, nuclear localization signal from simian virus 40 large T antigen; pA, poly A; hpi, hours post-injection. **(B)** Distribution of sgRNA target sites relative to H3K4me3 and H3K27ac ChIP-seq profiles at the *tyr* locus (chr15: 43798700–43801070 bp; danRer11). ChIP-seq (H3K4me3 and H3K27ac) datasets were obtained from Baranasic *et al.* (2022) [46]. **(C)** Box plots showing *tyr* mRNA expression at 48 hpi. Values represent mean ± SEM from six groups: wild-type, sgCtrl, and four sgRNAs targeting the *tyr* gene. Sample size (*n*) for each group is indicated below the boxes. Samples were collected from three independent injections, with each sample comprising a pool of five embryos. Different letters indicate statistically significant differences (*P* < 0.05, one-way ANOVA with Bonferroni’s post hoc test). sgCtrl, scrambled nontargeting control sgRNA.

We first assessed the ability of CRISPRi to repress gene expression *in vivo* using the *tyr* locus as a model. The optimized CRISPRi v2 construct, comprising dCas9 fused to a bipartite KRAB-MeCP2 repressor domain, was selected based on its strong silencing capacity in mammalian cells [21]. sgRNAs were designed to span −50 bp to +400 bp relative to the transcription start site (TSS) [30, 53], guided by ChIP-seq for H3K27ac and H3K4me3 peaks to ensure targeting of transcriptionally active and accessible chromatin regions (Fig. 1A and B).

qRT-PCR at 48 hpi revealed that all four sgRNAs (sgTyr1-4) significantly reduced *tyr* transcript abundance compared with scrambled controls (*P* < 0.05 by one-way ANOVA with Bonferroni correction), with sgTyr3 and sgTyr4 achieving the strongest repression (∼50% reduction; Fig. 1C). Consistent with these results, the most effective sgRNAs overlapped both open chromatin and active histone marks (Supplementary Fig. S1). An active Cas9-mediated knockout served as a positive control (Supplementary Fig. S2). These results confirmed that CRISPRi targeting of promoter–proximal sites within accessible, acetylated chromatin effectively silences transcription in zebrafish embryos. 

### Optimization of CRISPRi component ratios and concentrations for maximal repression with minimal toxicity

**Figure 2.**
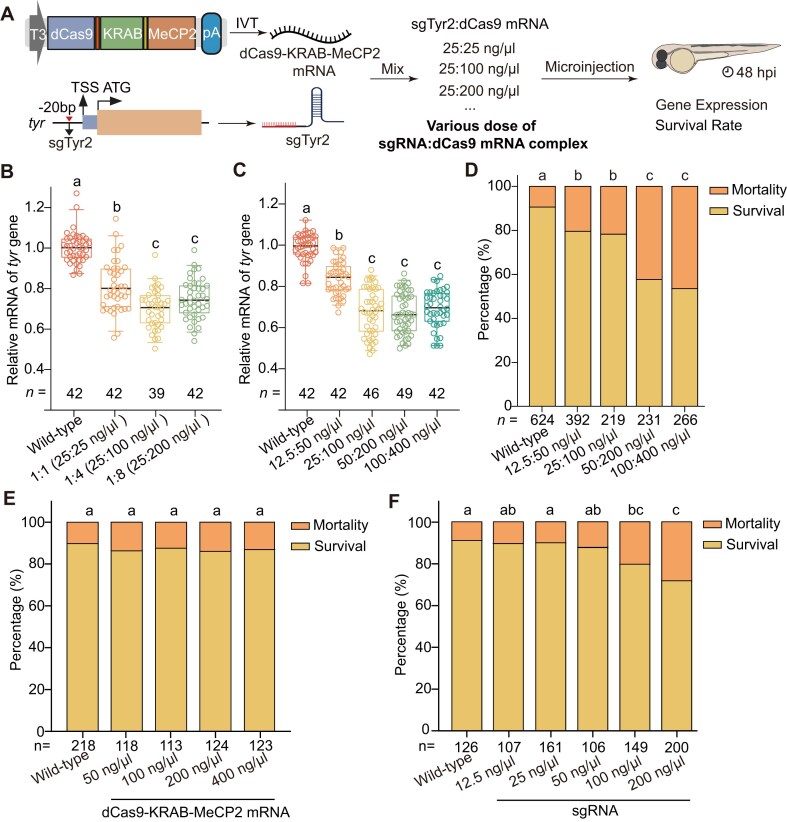
Optimization of CRISPRi system. (**A**) Schematic illustrating the optimization strategy for CRISPRi in zebrafish using mixtures of dCas9-KRAB-MeCP2 mRNA and sgRNA at varying doses. The sgRNA (sgTyr2) targeting the proximal promoter of the *tyr* gene was used in all experiments. Embryos were evaluated at 48 h post-injection (hpi) for target gene expression and survival rate. IVT, *in vitro* transcription. (**B** and **C**). Box plots showing *tyr* expression in zebrafish embryos at 48 hpf following co-injection of dCas9-KRAB-MeCP2 mRNA and sgTyr2 at different ratios (B) and final concentrations (C), alongside wild-type controls. Values represent mean ± SEM, with sample size (*n*) indicated below each box. Samples were collected from three independent injections, each consisting of a pool of five embryos. Statistical significance was determined by one-way ANOVA followed by Bonferroni-corrected pairwise comparisons; different letters indicate significant differences (adjusted *P* < 0.05). (D–F). Bar graphs showing embryo survival rates at 48 hpf following injection of (D) dCas9-KRAB-MeCP2 mRNA and sgTyr2 mixtures, (E) dCas9-KRAB-MeCP2 mRNA alone, or (F) sgTyr2 alone, at the indicated concentrations. Values represent mean from five independent injections, with total embryo number (*n*) indicated. Statistical significance was assessed by chi-square tests with post hoc Bonferroni correction. Different letters indicate significant differences (adjusted *P* < 0.005 in D and E; and adjusted *P* < 0.0033 in F).

We next optimized the ratio and concentration of CRISPRi components to balance silencing efficiency and embryo survival (Fig. 2A). Literature review indicated sgRNA:dCas9 mRNA ratios of 1:1 to 1:8 are commonly used in zebrafish CRISPR studies (Supplementary Table S2). Using sgTyr2, we tested ratios of 1:1 (25:25 ng/μl), 1:4 (25:100 ng/μl), and 1:8 (25:200 ng/μl). All ratios reduced *tyr* expression, but the 1:4 ratio achieved maximal repression (∼50% reduction) without additional benefit at 1:8 (Fig. 2B).

A concentration series at the 1:4 ratio (12.5:50, 25:100, 50:200, and 100:400 ng/μl) showed strongest silencing (∼53%) at 25:100 ng/μl, with no further improvement at higher doses (Fig. 2C). Survival rates were unaffected at 25:100 ng/μl but declined by ∼20% at ≥50:200 ng/μl (Fig. 2D).

To identify the source of toxicity, dCas9-KRAB-MeCP2 mRNA or sgRNA was injected at increasing concentrations. High sgRNA levels (200 ng/μl) significantly reduced survival (adjust *P* < 0.0033 by chi-square test with Bonferroni correction), whereas dCas9-KRAB-MeCP2 mRNA was well tolerated up to 400 ng/μl (Fig. 2E and F). We therefore selected 25:100 ng/μl (sgRNA:dCas9) as the optimal injection condition, providing robust repression with minimal developmental toxicity.

### CRISPRi-mediated repression of distal enhancers in globin locus

**Figure 3.**
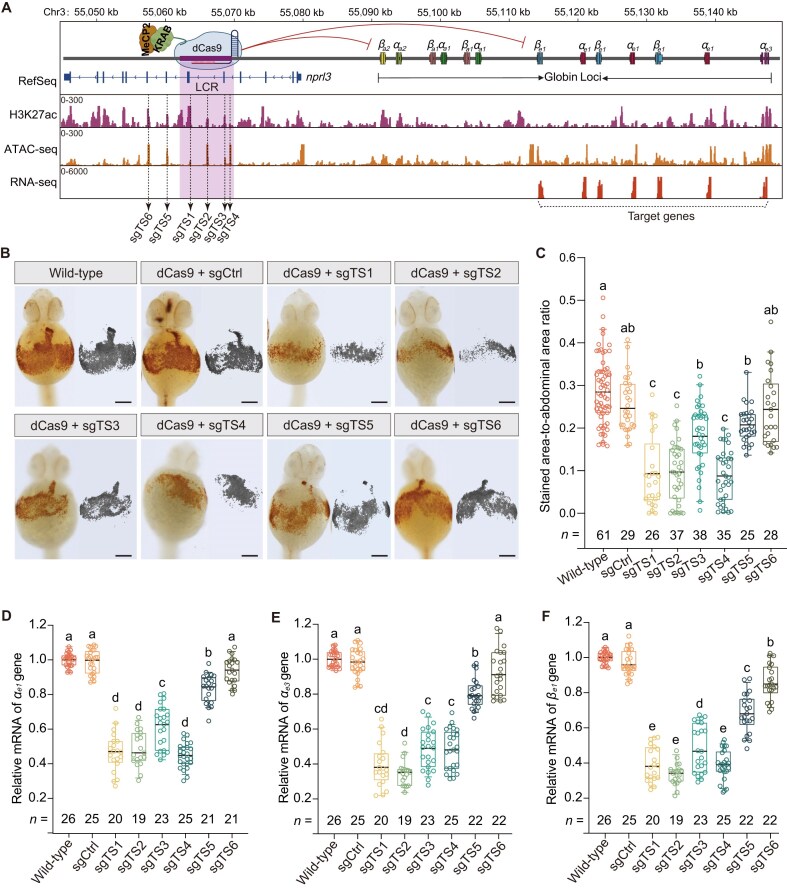
CRISPRi-mediated repression of globin genes by targeting distal enhancers. (**A**) ChIP-seq (H3K27ac), ATAC-seq, and RNA-seq profiles at the zebrafish globin locus (13 β-like globin genes) and the upstream LCR (purple shading, Chr3: 55044900–55 149100; danRer11). Dashed lines indicate sgRNA target sites: sgTS1–sgTS4 within the LCR and sgTS5–sgTS6 in H3K27ac/ATAC-seq peaks outside the LCR. A scrambled sgRNA (sgCtrl) served as a negative control. Based on RNA-seq signals, *β_e1_, α_e1_*, and *α_e3_* were selected as target genes for quantitative expression analysis. ATAC-seq and RNA-seq datasets were obtained from Franke *et al.* (2021) [45], and ChIP-seq for H3K27ac dataset was obtained from Baranasic *et al.* (2022) [46], respectively. (**B**) Representative o-dianisidine staining of the yolk in zebrafish embryos at 48 hpf following co-injection with dCas9-KRAB-MeCP2 mRNA with target-specific sgRNAs, alongside wild-type controls; scale bars: 100 μm. Each pattern was reproduced in three independent samples. (**C**) Quantification of erythrocyte abundance in embryos shown in (B), expressed as the ratio of *o*-dianisidine–positive area to total yolk area (ImageJ). Values represent mean ± SEM, with total embryo number (*n*) from three independent injections indicated. Statistical significance was assessed by one-way ANOVA with Bonferroni correction; different letters denote significant differences (*P* < 0.05). sgCtrl, scrambled non-targeting control sgRNA. (**D–F**) Relative expression of *α_e1_* (D), *α_e3_* (E), and *β_e1_* (F) at 48 hpf after co-injection with dCas9-KRAB-MeCP2 mRNA and target-specific sgRNAs. Values represent mean ± SEM, with sample size (*n*) indicated below each box. Samples were collected from three independent injections, each consisting of a pool of five embryos. Statistical significance was determined by one-way ANOVA with Bonferroni correction; different letters indicate significant differences (adjusted *P* < 0.05). sgCtrl, scrambled nontargeting control sgRNA.

We next applied the optimized CRISPRi system to study distal enhancer function *in vivo*. The zebrafish globin locus is regulated by a well-characterized LCR located ∼40 kb upstream, which influences 13 β-like globin genes (*β_a2_, α_a2_, β_a1a/b_, α_a1a/b_, β_e1a/b/c_, α_e1a/b/c_*, and *α_e3_*). ATAC-seq, H3K27ac ChIP-seq, and RNA-seq identified multiple accessible regions within the LCR with enhancer-like signatures (Fig. 3A). Based on these signals, we designed four sgRNAs (sgTS1 to sgTS4) targeting the LCR (Fig. 3A). We also designed two sgRNAs (sgTS5 and sgTS6) outside the LCR but within H3K27ac/ATAC-seq peaks (Fig. 3A). A scrambled sgRNA (sgCtrl) served as a negative control.

At 48 hpf, *o*-dianisidine staining revealed significant reductions in hemoglobin production and erythrocyte formation in embryos injected with sgTS1–sgTS4 and sgTS5 compared to controls (Fig. 3B and C; Supplementary Figs S3 and S4). Within the LCR, sgTS1, sgTS2, and sgTS4 produced the strongest phenotypic effects, followed by sgTS3, whereas sgTS5 (near the LCR flank) had a milder effect, and sgTS6 (further from the LCR) showed no significant change (Fig. 3C).

qRT-PCR analysis (≥19 embryos from three independent biological replicates per group) confirmed that repression of LCR-associated sites led to significant downregulation of embryonic globin genes (*α_e1_, α_e3,_* and *β_e1_*), with knockdown efficiencies reaching ∼80% for the strongest targets (*P* < 0.05 by one-way ANOVA with Bonferroni correction; Fig. 3D–F). The transcriptional reductions closely mirrored the severity of hemoglobin phenotypes, underscoring a strong correlation between enhancer repression, globin gene expression, and blood cell development. Together, these results confirm the LCR’s central role in regulating globin expression and validate CRISPRi as a tool for *in vivo* enhancer perturbation. 

### Genome-wide identification of enhancer–promoter interactions in zebrafish embryos

**Figure 4.**
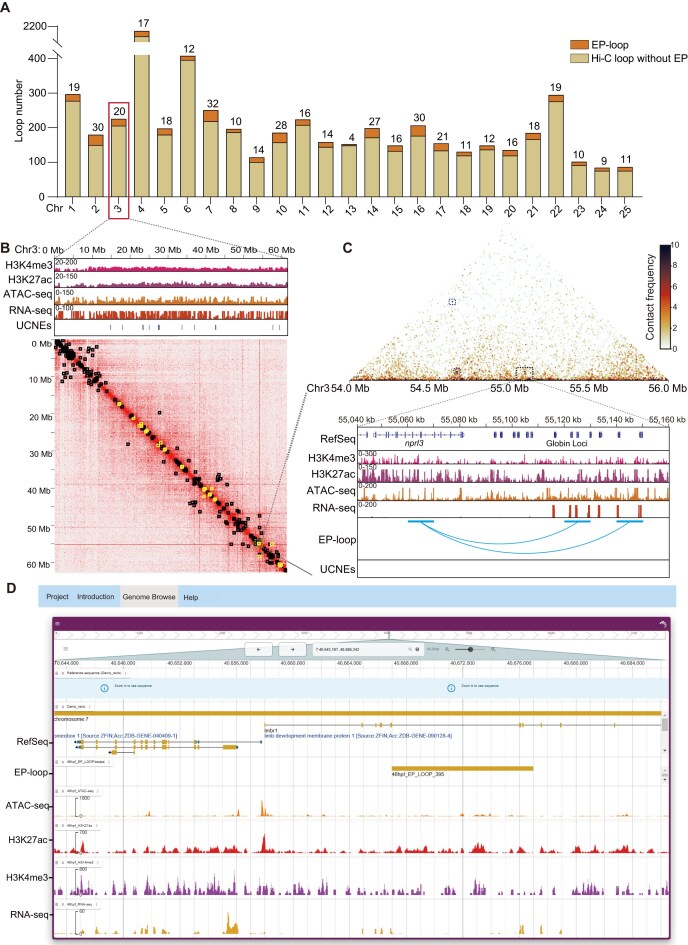
Genome-wide identification of EP interactions in zebrafish embryos at 48 hpf. (**A**) Bar plots showing the distribution of Hi-C loops across zebrafish chromosomes, classified by the presence (orange) or absence (light yellow) of EP interactions, as defined by histone modification and chromatin accessibility profiles. The number of EP loops per chromosome is indicated above each bar. (**B**) Hi-C contact map of chromosome 3 (Chr3; lower panel), with contact intensity represented by a heatmap. The upper panel shows ChIP-seq (H3K4me3, H3K27ac), ATAC-seq, RNA-seq, and UCNEs. Hi-C loops (black squares) and EP loops (yellow squares) are marked at stripe intersections. (**C**) Hi-C map of a 2-Mb region on Chr3 (54–56 Mb) at 10-kb resolution. Three EP loops are highlighted, linking globin gene promoters to the LCR (blue arcs). ChIP-seq (H3K4me3, H3K27ac), ATAC-seq, RNA-seq, EP-loop arcs, and UCNE tracks are shown for the LCR and adjacent globin locus regions.ATAC-seq and RNA-seq datasets were obtained from Franke *et al.* (2021) [45], and ChIP-seq (H3K4me3, H3K27ac) datasets were obtained from Baranasic *et al.* (2022) [46], respectively. (**D**) Screenshot of the interactive web portal (http://121.40.142.177/), which provides 48-hpf zebrafish genomic data including gene annotations, expression profiles, multi-omics tracks, and EP-loop information.

To systematically map interactions between *cis-*regulatory elements and target gene promoters, we performed Hi-C assay on 48 hpf zebrafish embryos. Our Hi-C analysis identified 6535 potential interaction pairs distributed across all chromosomes, with most chromosomes containing 150–400 pairs. Notably, chromosome 4 (Chr4) contained an unusually high number of loops (2185 pairs, Fig. 4A), consistent with previous reports and likely linked to its heterochromatin-rich, epigenetically distinctive landscape [28, 54]. By integrating H3K4me3 (promoter marker), H3K27ac (enhancer marker), and ATAC-seq signals, we identified 434 effective EP loops (EP-loops) across the zebrafish genome (Fig. 4A and Supplementary Table S3).

On chromosome 3 (Chr3), the Hi-C contact map displayed typical topological structures and extensive chromatin interactions (black squares, Fig. 4B and Supplementary Fig. S5). Additionally, 20 specific EP-loops were identified based on combined multi-omics signals (yellow squares, Fig. 4B and Supplementary Fig. S5). Notably, the distal enhancers within the 54–56 megabases (Mb) region on Chr3, experimentally validated to regulate expression of the zebrafish globin locus (as shown in Fig. 3A), were also detected in our EP-loop analysis (Fig. 4C), demonstrating the reliability of our multi-omics approach.

Furthermore, an interactive web portal (http://121.40.142.177/) was developed to detail the content and significance of each omics data in the present study (including gene location, expression, multi-omics signals, and spatial interaction information), thereby enhancing the user’s understanding and interaction with the multi-omics data presented (Fig. 4D). 

### Validation of known and novel developmental enhancers using CRISPRi

**Figure 5.**
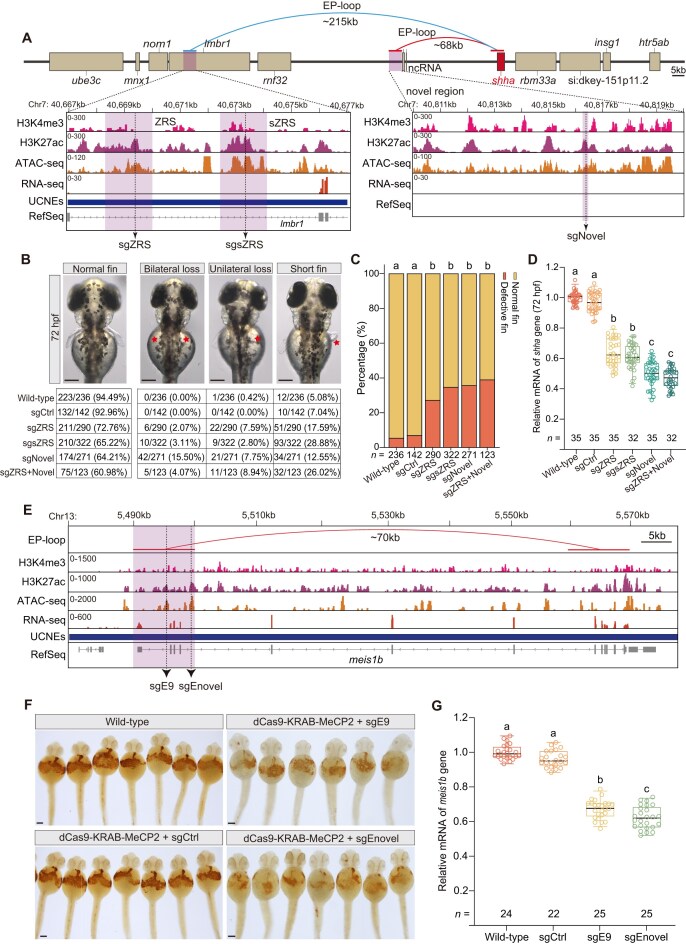
Functional validation of novel enhancers in zebrafish development using *in vivo* CRISPRi. (**A**) Genomic organization of the zebrafish *shha* locus. EP-loop analysis identified two known enhancers—the ZRS (blue arc) and its shadow enhancer (sZRS, blue arc)—as well as a novel enhancer ∼68 kb upstream of *shha* (red arc). Tracks show EP loops, ChIP-seq (H3K4me3, H3K27ac), ATAC-seq, RNA-seq, and UCNEs across three regulatory regions (purple shading). Dashed lines mark sgRNA target sites. (**B**) Representative images (top) and summary table (bottom) of pectoral fin phenotypes at 72 hpf following injection of dCas9-KRAB-MeCP2 mRNA with single-sgRNAs (sgZRS, sgsZRS, and sgNovel) or dual-sgRNAs (sgZRS + sgNovel). Defective phenotypes included short fin, unilateral loss, and bilateral loss (red asterisks). sgCtrl, scrambled nontargeting control sgRNA; scale bars: 200 μm. (**C**) Quantification of defective fin phenotypes shown in (B). Data represent mean of the total embryos (*n*) from three independent injections. Statistical comparisons were performed by chi-square test with Bonferroni’s post-hoc test; different letters indicate significant differences (adjusted *P* < 0.0033). (**D**) Box plots of *shha* expression at 72 hpf following injection of single- or dual-sgRNAs targeting the enhancers in (A). Values are relative to wild type, with each point representing a pool of five embryos from one of three independent injections. Statistical significance was determined by one-way ANOVA with Bonferroni correction; different letters indicate significant differences (adjusted *P* < 0.05). sgCtrl, scrambled nontargeting control sgRNA. (**E**) Genomic organization of the zebrafish *meis1b* locus, highlighting the known enhancer E9 and a novel enhancer (Enovel) from the EP-loop list. Tracks include EP loops, ChIP-seq (H3K4me3, H3K27ac), ATAC-seq, RNA-seq, and UCNEs. Dashed lines mark sgRNA target sites. ATAC-seq and RNA-seq datasets were obtained from Franke *et al.* (2021) [45], and ChIP-seq (H3K4me3, H3K27ac) datasets were obtained from Baranasic *et al.* (2022) [46], respectively. (**F**) Representative *o*-dianisidine staining images showing hemoglobin production and erythrocyte formation at 48 hpf following injection of sgRNAs targeting E9 or Enovel, alongside wild-type controls. sgCtrl, scrambled non-targeting control sgRNA; scale bars: 100 μm. (**G**) Box plots of *meis1b* expression at 48 hpf following enhancer targeting as in (E). Data and statistical testing as in (D).

To functionally validate *cis-*regulatory elements identified from our Hi-C-based EP loop list, we used CRISPRi to target selected long-range interactions. Among these, the ZRS and its shadow enhancer sZRS—known regulators of *shha* [7]—were located ∼215 kb upstream of *shha* (Fig. 5A). Our EP-loop analysis also revealed a novel enhancer ∼68 kb upstream of *shha*. We designed three sgRNAs targeting ZRS, sZRS, and this novel element, along with a scrambled sgRNA (sgCtrl) as a negative control (Fig. 5A). At 72 hpf, morphological analysis showed pectoral fin-ray defects in 27.26–39.02% of embryos injected with sgRNAs against ZRS, sZRS, the novel element, or both ZRS and the novel element (Fig. 5B and Supplementary Fig. S6). The novel element produced the highest proportion of fin-loss phenotypes (Fig. 5B and C), correlating with the strongest repression of *shha* expression (Fig. 5D), a key driver of pectoral fin development. Moreover, dual-sgRNA targeting of both ZRS and the novel enhancer produced the most pronounced fin defects and *shha* repression. However, these effects were not significantly greater than those from single-sgRNA targeting of the novel enhancer alone (Fig. 5B–D), suggesting limited additive functional impact.

To evaluate the specific contribution of the KRAB-MeCP2 repressor module, we generated a truncated CRISPRi construct containing only dCas9 (Supplementary Fig. S7A). Co-injection of dCas9 alone mRNA with the novel element sgRNA reduced *shha* expression modestly, consistent with steric hindrance, whereas dCas9-KRAB-MeCP2 achieved substantially stronger repression (adjust *P* < 0.05 by one-way ANOVA with Bonferroni correction; Supplementary Fig. S7B).

We next examined the *meis1b* locus, essential for hematopoiesis [55]. Alongside the known enhancer E9 [55], we identified a novel enhancer (Enovel) ∼70 kb downstream of *meis1b* (Fig. 5E). Targeting E9 or Enovel with sgRNAs significantly reduced blood cell formation, as measured by *o*-dianisidine staining (Fig. 5F and Supplementary Fig. S8), and downregulated *meis1b* mRNA levels by qRT-PCR (Fig. 5G). Notably, Enovel caused stronger transcriptional repression than E9 (Fig. 5G). 

**Figure 6.**
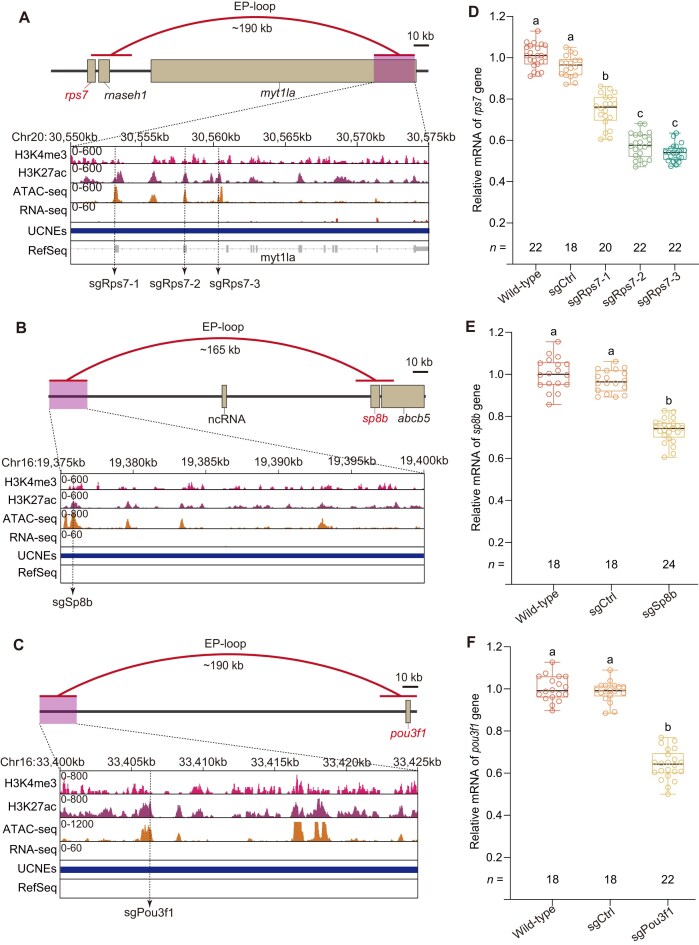
CRISPRi-mediated repression of genes via disruption of long-range EP interactions. (**A–C**) Genomic organization of the zebrafish *rps*7 (A), *sp8b* (B), and *pou3f1* (C) loci. Tracks show EP loops (red arcs; distances annotated), ChIP-seq (H3K4me3, H3K27ac), ATAC-seq, RNA-seq, and UCNEs, with potential enhancer regions shaded in purple. Dashed lines indicate sgRNA target sites within predicted enhancer regions. ATAC-seq and RNA-seq datasets were obtained from Franke *et al.* (2021) [45], and ChIP-seq (H3K4me3, H3K27ac) datasets were obtained from Baranasic *et al.* (2022) [46], respectively. (**D–F**) Box plots showing relative expression levels of *rps7* (D), *sp8b* (E), and *pou3f1*  **(F)** at 48 hpf following injection of dCas9-KRAB-MeCP2 mRNA with target-specific sgRNAs. Values are normalized to wild-type controls and represent mean ± SEM, with sample size (*n*) indicated below each box. Samples were collected from three independent injections, each consisting of a pool of five embryos. Statistical comparisons were performed using one-way ANOVA with Bonferroni’s post-hoc test; different letters indicate significant differences (adjusted *P* < 0.05). sgCtrl, scrambled nontargeting control sgRNA.

Beyond these well-characterized developmental loci, we tested three additional EP pairs from our EP-loop list (Fig. 6A–C). Targeting these enhancers led to significant downregulation of their predicted target genes (Fig. 6D–F). 

Together, these results establish direct functional links between multiple novel enhancers and their developmental roles, demonstrating that integrating multi-omics EP-loop mapping with *in vivo* CRISPRi perturbation provides a powerful framework for dissecting *cis-*regulatory control in zebrafish embryos.

## Discussion

Noncoding *cis-*regulatory elements, especially enhancers, are crucial for understanding gene expression and complex traits, given their fundamental roles in evolution, development, and disease [3, 4]. These elements drive diverse biological processes and have far-reaching implications for studies of development, pathology, and evolutionary biology [56, 57]. Traditional methods for studying non-coding regions often involve deletion-based approaches, which are time-intensive, requiring multiple generations to achieve homozygosity, and may not precisely capture enhancer-specific functions [58]. With advancements in CRISPR technology, CRISPRi offers a promising alternative by inhibiting gene expression without DNA cleavage, thereby preserving coding sequences while blocking putative transcription factor binding sites. This approach enables targeted functional analysis of enhancers without altering genomic integrity [59].

Zebrafish offer an ideal model for the development and disease studies due to their *ex vivo* embryonic development, transparent and easily observable early-stage phenotypes, and widely used in blood, heart development and related diseases. It is also a valuable model for high-throughput screening for the drug development and the discovery of new therapeutics for human disease [60]. In this study, we optimized the CRISPRi system in zebrafish, fine-tuning key parameters such as concentration and component ratios to establish a reliable and broadly applicable protocol (Fig. 2). To enable versatility, we used mRNA rather than protein for delivering dCas9-KRAB-MeCP2 (Figs 1A and 2A), allowing straightforward incorporation of future activators or repressors, such as the catalytic domains of DNA methyltransferases (e.g. Dnmt3A and Dnmt3L) or transcriptional activators (e.g. Viral Protein 64 (VP64), SunTag). This approach facilitates scalable mRNA production for efficient microinjection into embryos and enables high-throughput applications. By combining CRISPRi/a with an sgRNA library, this system holds potential as a powerful tool for generating disease models, validating novel drug targets, and discovering new therapeutics.

Despite CRISPRi’s advantages for functional annotation of non-coding elements, its use in zebrafish has been limited, primarily focusing on targeting proximal promoter regions without significant exploration of enhancer functions [61, 62]. This gap is likely due to the lack of comprehensive enhancer location data. Previous approaches, such as genetic reporter lines fused with fluorescent proteins, have been challenging for narrowing enhancer regions and scaling up for large-scale identification. In this study, we combined multi-omics datasets, including Hi-C interactions, ATAC-seq (open chromatin), H3K27ac (enhancer marker), and H3K4me3 (promoter marker), to systematically map genome-wide EP interactions in a high-throughput manner. By integrating multi-omics data, we identified 434 EP loops across the genome (Fig. 4A and Supplementary Table S3), creating a valuable resource for gene regulation studies, accessible through a user-friendly web portal (http://121.40.142.177/) we developed (Fig. 4D).

Interestingly, Hi-C analysis revealed an exceptionally high loop density on Chr4, suggesting unique regulatory and structural specialization during early embryogenesis. A key feature of Chr4 is the large miR-430 cluster, which produces the earliest and most abundant zygotic transcripts responsible for maternal mRNA clearance during zygotic genome activation [63]. During this transition, the repetitive miR-430 locus undergoes extensive chromatin reorganization, shifting from a heterochromatic to a transcriptionally active domain [54]. Owing to its repetitive architecture, this region can be efficiently targeted by pairs of sgRNAs that recognize multiple miR-430 copies simultaneously, a strategy previously validated for this locus [64]. This design provides a distinct advantage for achieving coordinated perturbation of the entire miR-430 array using CRISPRi. Beyond miR-430, Chr4 also contains extensive zinc-finger (ZnF) gene clusters and transposable element-derived repeats that likely reinforce chromatin insulation and promote long-range interactions [65, 66], maintaining its loop-rich topology even after transcription subsides. The dense looping architecture of Chr4 may thus represent a persistent topological scaffold established during early genome activation to ensure robust and coordinated developmental gene regulation. Nonetheless, the high repeat content of Chr4 could partly inflate loop estimates due to mapping and loop-calling challenges in repetitive regions. Integrating additional omics layers, including chromatin accessibility, histone modification, and transcriptional interaction data, will help refine promoter-enhancer assignments. Consistently, our 434 EP loops exhibited even chromosomal distribution, supporting the robustness of our analysis.

As a proof of concept, we tested two well-defined developmental processes—blood cell and fin formation—to validate predicted EP relationships. For known enhancers, such as LCR region of globins [5, 6] and E9 region of *meis1b* in hematopoiesis [55], ZRS and sZRS of *shha* in fin development [7], CRISPRi-mediated repression produced the expected regulatory effects (Figs 3B–F, 5B–D, and 5F–G; Supplementary Figs S3 and S6), confirming our EP loop predictions (Figs 3A, and 5A and E). We also identified novel enhancers with even stronger impacts on fin and blood development, providing new insights into regulatory pathways underlying organogenesis and hematologic disorders. In the *shha* locus, both the canonical ZRS (∼215 kb upstream) and a newly identified enhancer 68 kb upstream drive fin expression (Fig. 5A), but dual repression did not result in strictly additive effects (Fig. 5B–D). This likely reflects redundant or hierarchical enhancer organization within a shared EP hub, a principle commonly seen in developmental loci where shadow enhancers provide robustness rather than linear additivity [67, 68]. Moreover, KRAB-MeCP2-based CRISPRi can induce heterochromatin spreading, which may disrupt the local regulatory architecture and contribute to the sub-additive phenotype. Our Hi-C data place both enhancers within the same TAD, consistent with a model in which the proximal enhancer provides dominant activation while ZRS ensures transcriptional buffering during fin morphogenesis.

While reproducible and versatile, our zebrafish CRISPRi system currently mediates moderate transcriptional repression (~40%–60%), consistent with KRAB-based repression efficiencies in mammalian systems [21, 59, 69]. This limitation likely arises from the intrinsic difficulty of recruiting dCas9-KRAB-MeCP2 to compact or rapidly remodeling chromatin during early embryogenesis. Although partial repression may limit applications requiring complete silencing, it offers distinct advantages for analyzing essential or dosage-sensitive developmental genes, where full knockout is often lethal. This tunable modulation allows quantitative interrogation of gene activity and graded phenotypic outcomes. Future refinements, including improved repressor domains or inducible CRISPRi systems [70], may further enhance the dynamic range and precision of transcriptional control *in vivo*.

Our study therefore establishes a scalable CRISPRi-based enhancer mapping strategy in zebrafish, providing a versatile platform to explore enhancer functions in development and evolution. By integrating enhancer annotation with comparative genomics, this approach enables the identification of conserved enhancers essential for development and lineage-specific enhancers driving phenotypic adaptations [71]. For example, enhancers regulating traits like fin or blood cell development offer insights into how non-coding elements contribute to evolutionary innovations. The optimized CRISPRi system allows *in vivo* perturbation of enhancer activity, enabling direct functional validation of conserved and species-specific regulatory elements. This framework bridges computational predictions with experimental validation, linking enhancer variation to phenotypic diversity and adaptive evolution in vertebrates.

Despite extensive optimization of sgRNA:dCas9 ratios, total mRNA concentrations, and toxicity controls, CRISPRi-mediated perturbations in zebrafish embryos produced variable phenotypic outcomes, particularly in fin development (Fig. 5B and C). This variability likely reflects both technical and biological factors. Targeting heterogeneity—arising from differences in sgRNA binding efficiency, natural sequence variation, and mosaic gene silencing—can yield inconsistent repression across cells [30, 72]. Moreover, zebrafish exhibit strong developmental buffering through maternal mRNA reserves, paralog redundancy, and compensatory regulatory networks, which can mask morphological effects unless expression falls below a critical threshold [73, 74]. For instance, partial *shha* repression may be offset by *tbx5* and *hox* gene activity [75, 76]. Intrinsic embryo-to-embryo variation further contributes to incomplete penetrance [77]. These findings highlight the necessity of integrating precise molecular measurements with morphological phenotyping to accurately define *cis-*regulatory element function *in vivo*.

In summary, this study establishes a comprehensive framework for characterizing *cis-*regulatory elements in zebrafish through multi-omics data integration and CRISPRi-based functional perturbation. The resulting EP map and optimized CRISPRi methodology provide a flexible and scalable platform for dissecting non-coding regulation in development, disease, and evolution. By linking chromatin architecture to gene expression and phenotype, this work lays the foundation for systematic functional annotation of the noncoding genome in vertebrates.

## Supplementary Material

gkaf1367_Supplemental_File

## Data Availability

The raw sequencing data of Hi-C in our study have been deposited in the Genome Sequence Archive in BIG Data Center (https://bigd.big.ac.cn/), Beijing Institute of Genomics (BIG), Chinese Academy of Sciences, under the BioProject number: PRJCA049852. The analysis source code underlying the final version of the paper is available to download from Zenodo at https://doi.org/10.5281/zenodo.16919346.
