# Supplementary material for: Systematic enhancer mapping and functional analysis in zebrafish with optimized CRISPR interference
Source: Nucleic Acids Res. 2025 Dec 18;53(22):gkaf1367. doi: 10.1093/nar/gkaf1367 (PMC12714565; doi:10.1093/nar/gkaf1367)
Supplement: gkaf1367_Supplemental_File [file gkaf1367_supplemental_file.docx]

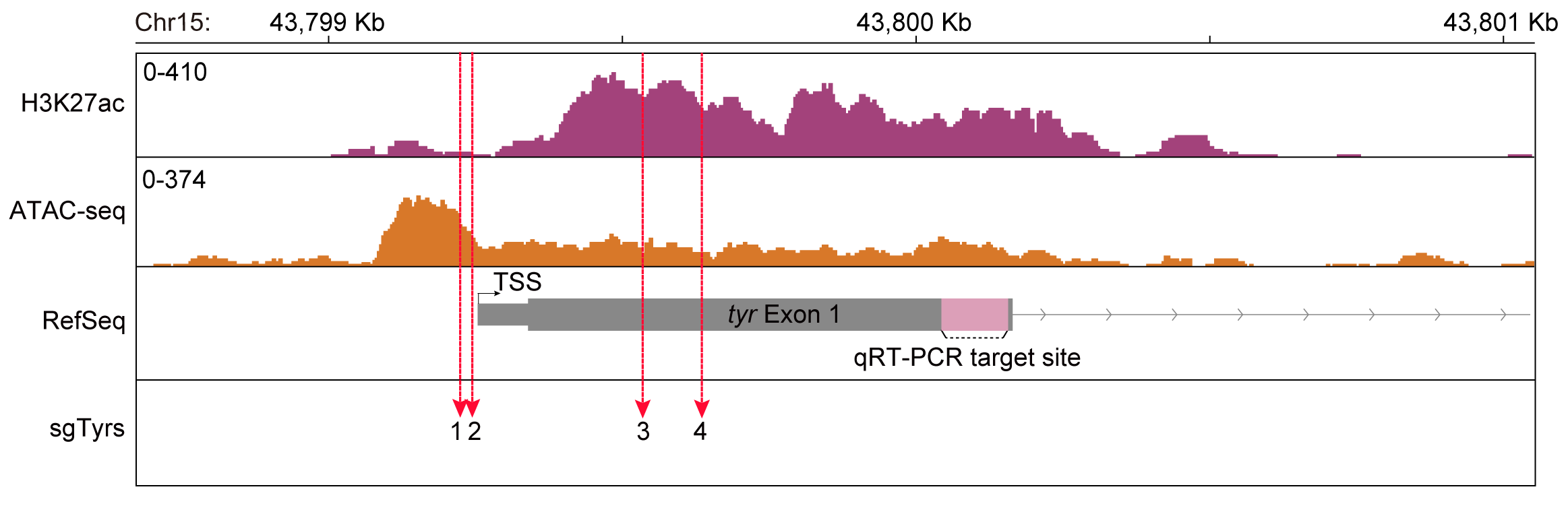


**Fig. S1** **|** Distribution of sgRNA target sites relative to ATAC-seq and H3K27ac ChIP-seq profiles at the *tyr* locus (Chr15: 43,798,700-43,801,070 bp; danRer11). ATAC-seq and ChIP-seq for H3K27ac datasets were obtained from Franke et al., 2021 and Baranasic et al., 2022, respectively.


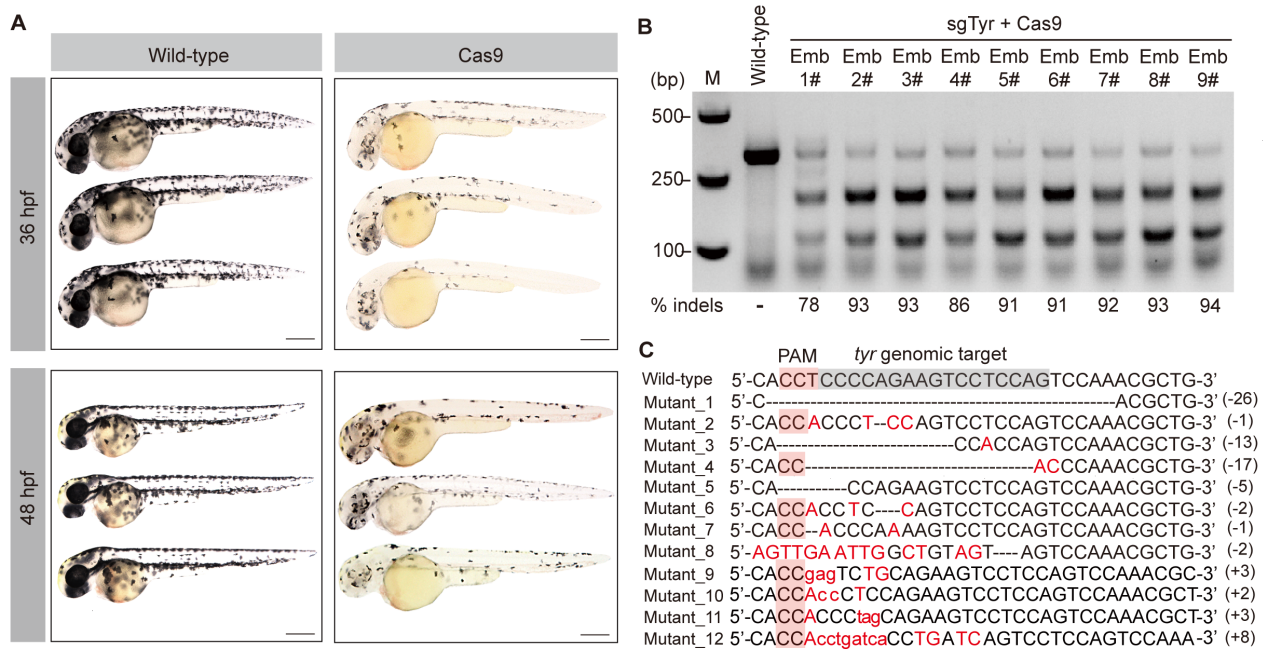


**Fig. S2 | CRISPR/Cas9-mediated mutagenesis of the *tyr* gene efficiently induces indels in zebrafish embryos.**

1. Representative images of melanin deposition in zebrafish embryos at 36 and 48 hpf following co-injection with Cas9 and sgTyr targeting the *tyr* gene, alongside wild-type controls. Scale bars, 200 μm.

**B.** T7E1 nuclease assay of PCR amplicons spanning the sgTyr target site in nine individual editing embryos, alongside wild-type controls. Mutation efficiency was assessed by calculating the ratio of the intensity of the cleaved bands to the total intensity of all bands after enzyme digestion. High indel efficiencies (78-94%) were observed across these injected embryos (lanes 3-11). M: DNA ladder.

**C.** Sanger sequencing and sequence alignment of cloned amplicons from injected embryos. Twelve representative mutant alleles (Mutant_1 - Mutant_12) are shown below the wild-type reference sequence. Deleted bases are indicated by dashes (-), inserted bases are shown in lowercase red letters, and base mutations are highlighted in uppercase red letters. The corresponding net length changes are indicated in parentheses to the right. The original protospacer-adjacent motif (PAM) is highlighted with a red background, and the *tyr* target site is shown with a gray shading.


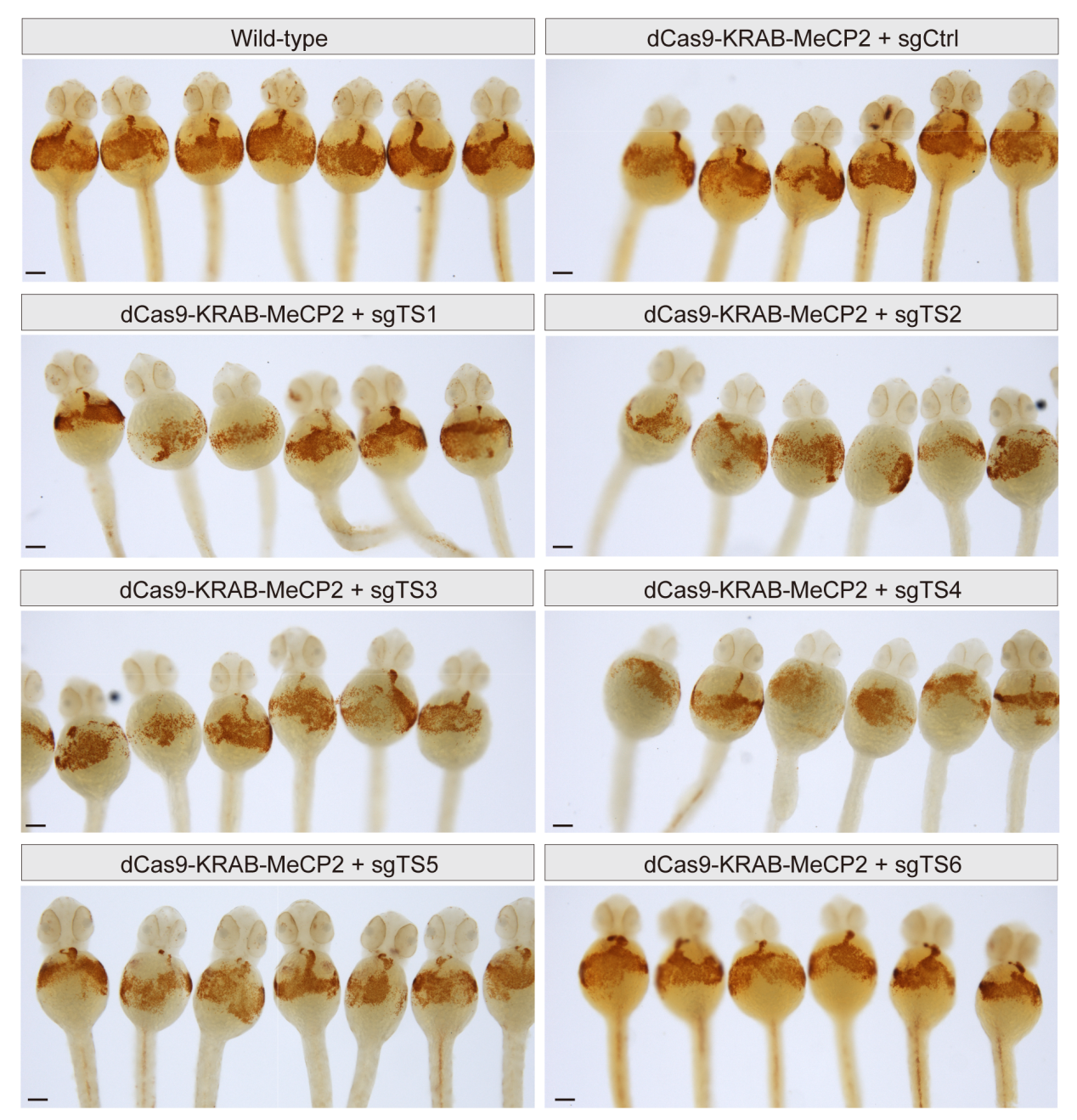


**Fig. S3 | Erythrocyte formation is affected in CRISPRi components injected embryos.** O-dianisidine staining was used to detect the distribution and number of hemoglobin-positive cells on the yolk sac of zebrafish embryos at 48 hpf following injection of sgRNAs targeting the LCR or its flanking regions of globin genes (sgTS1 - sgTS4 targeting the LCR, and sgTS5, sgTS6 targeting LCR flanking regions), alongside wild-type controls. Scale bars: 100 μm. sgCtrl, non-targeting scambled control sgRNA.


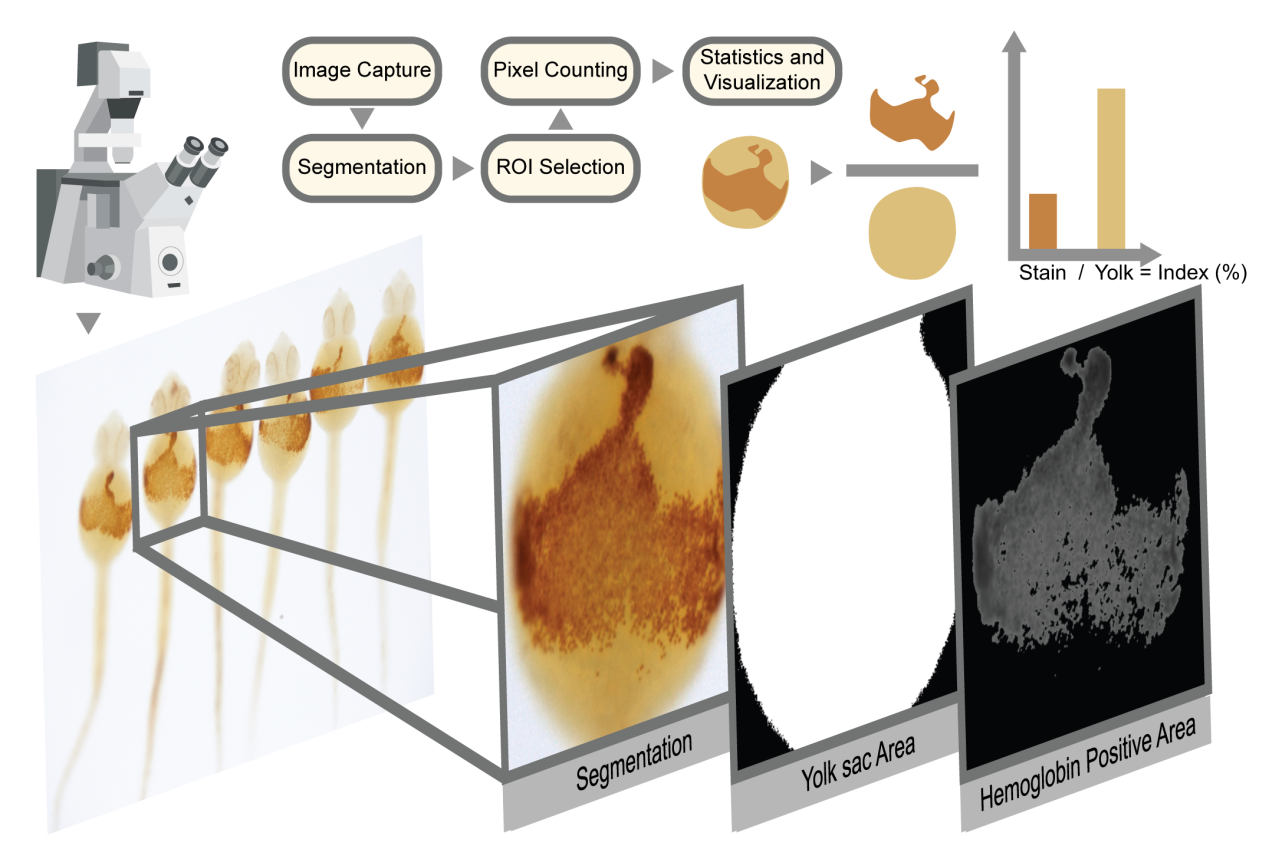


**Fig. S4 | Workflow for quantifying hemoglobin-positive signal.** The yolk sac was first manually segmented from each image to generate a binary yolk mask. Within this mask, background and hemoglobin-negative yolk regions were excluded by creating separate negative masks. Hemoglobin-positive pixels were then identified and counted alongside the hemoglobin-negative pixels. The hemoglobin index was calculated as the ratio of hemoglobin-positive pixel count to total yolk pixel count.


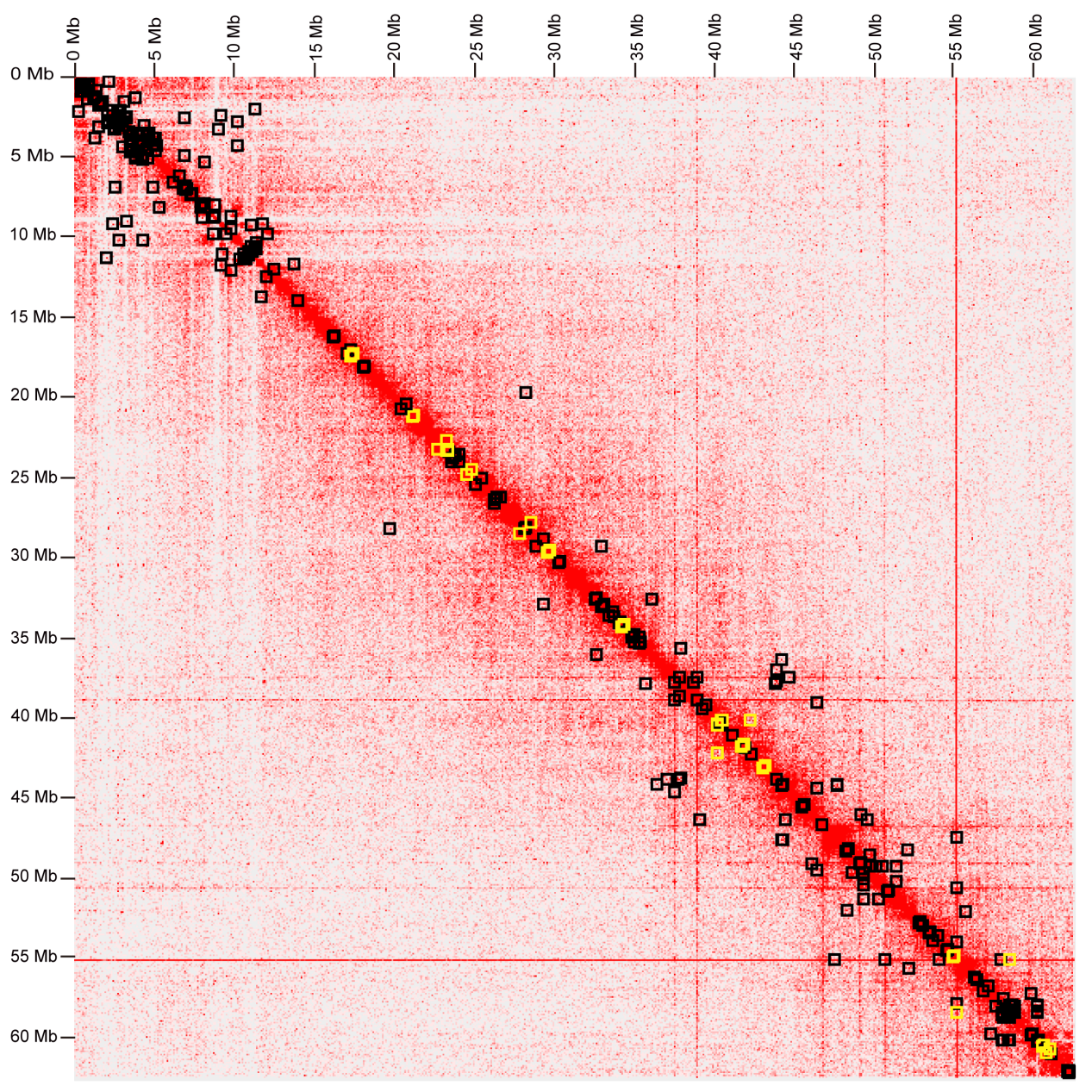


**Fig. S5 | Hi-C contact map of chromosome 3 (Chr3).** Contact intensity is represented by a heatmap. Hi-C loops (black squares) and EP loops (yellow squares) are marked at stripe intersections.


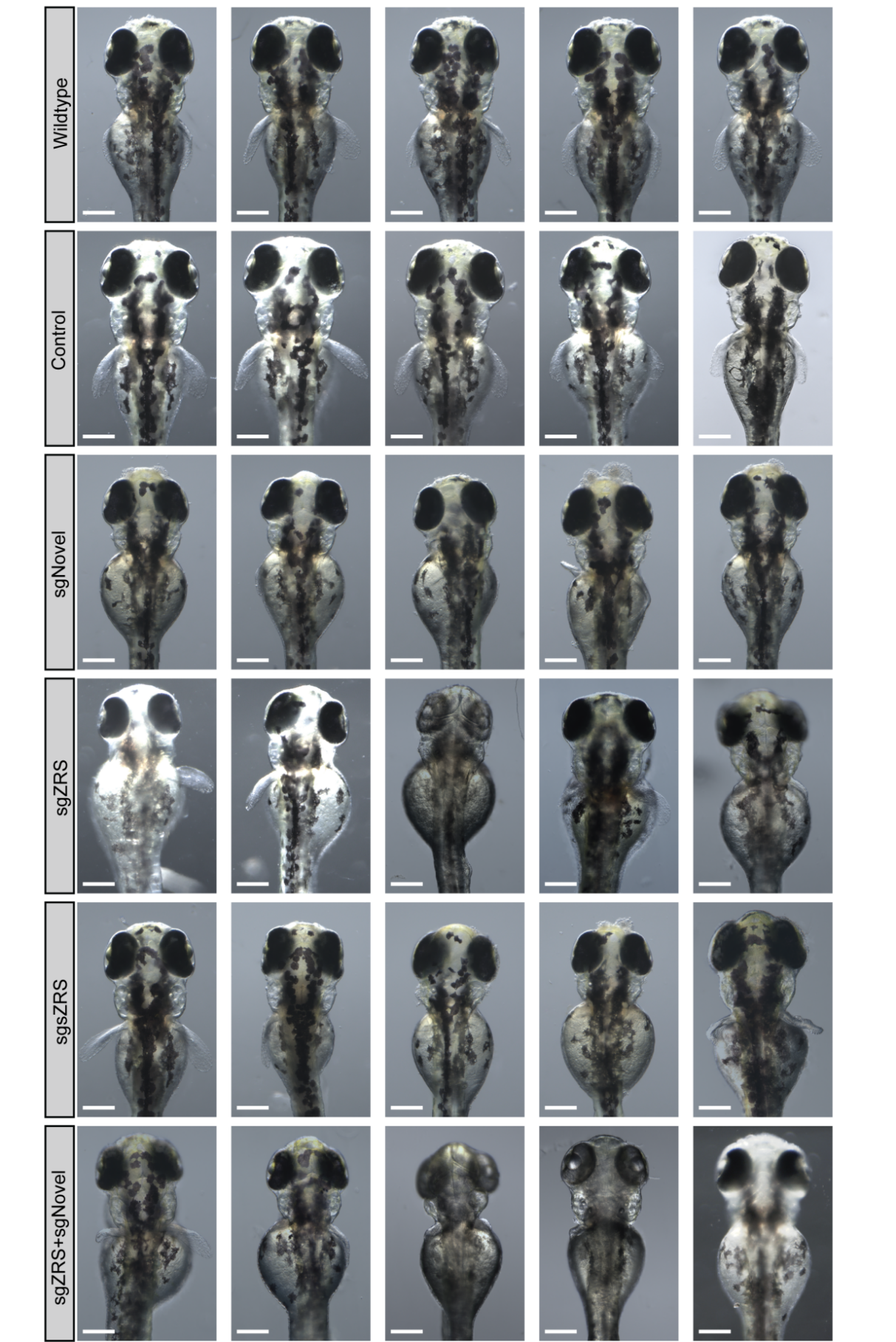


**Fig. S6 | CRISPRi-mediated pectoral fin developmental defects in zebrafish embryos.** Representative images of pectoral fin phenotypes at 72 hpf following injection of dCas9-KRAB-MeCP2 mRNA with single-sgRNAs (sgZRS, sgsZRS, sgNovel) or dual-sgRNAs (sgZRS + sgNovel). Defective phenotypes included short fin, unilateral loss, and bilateral loss. sgCtrl, scrambled non-targeting control sgRNA. Scale bars: 200 μm.


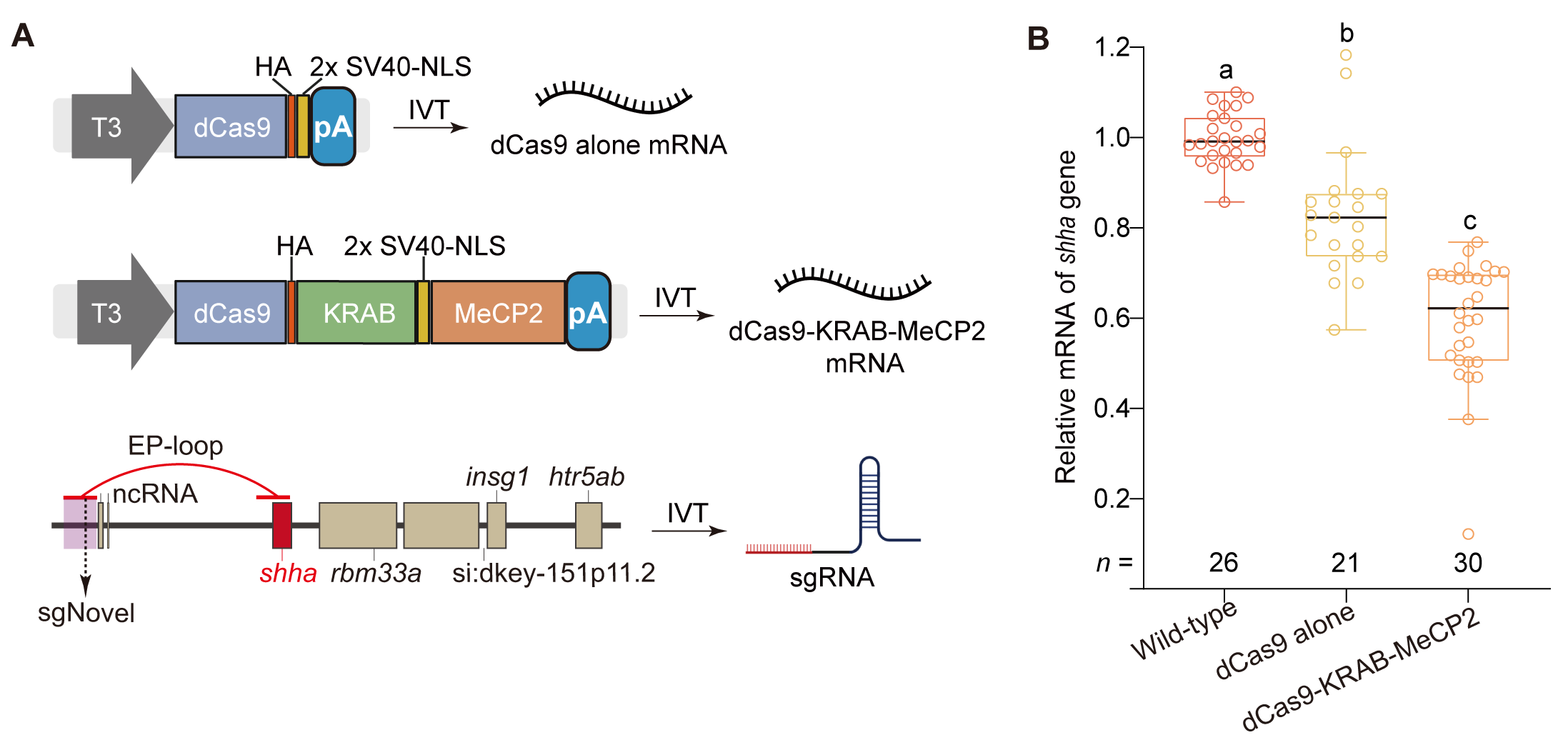


**Fig. S7 | dCas9-KRAB-MeCP2 mediated stronger repression of *shha* than dCas9 alone.**

1. Schematic of the different nuclease-inactive Cas9 (dCas9) mRNA used to repress *shha* expression. dCas9 alone (top) and Krüppel-associated box (KRAB)-methyl-CpG-binding protein 2 (MeCP2) fused dCas9 (dCas9-KRAB-MeCP2) co-injected as *in vitro* transcribed mRNA with the sgRNA (sgNovel; bottom) targeting the distal *shha* enhancer. HA, human influenza hemagglutinin epitope tag; SV40-NLS, nuclear localization signal from simian virus 40 large T antigen; IVT, *in vitro* transcription; pA, poly A.
2. Box plots of *shha* expression at 72 hpf following co-injection of dCas9-KRAB-MeCP2, or dCas9 alone mRNA with sgNovel targeting the *shha* enhancer. Values are normalized to wild-type controls and represent mean ± SEM, with sample size (*n*) indicated below each box. Samples were collected from three independent injections, each consisting of a pool of five embryos. Statistical comparisons were performed using one-way ANOVA with Bonferroni’s post-hoc test; different letters indicate significant differences (*P* < 0.05).


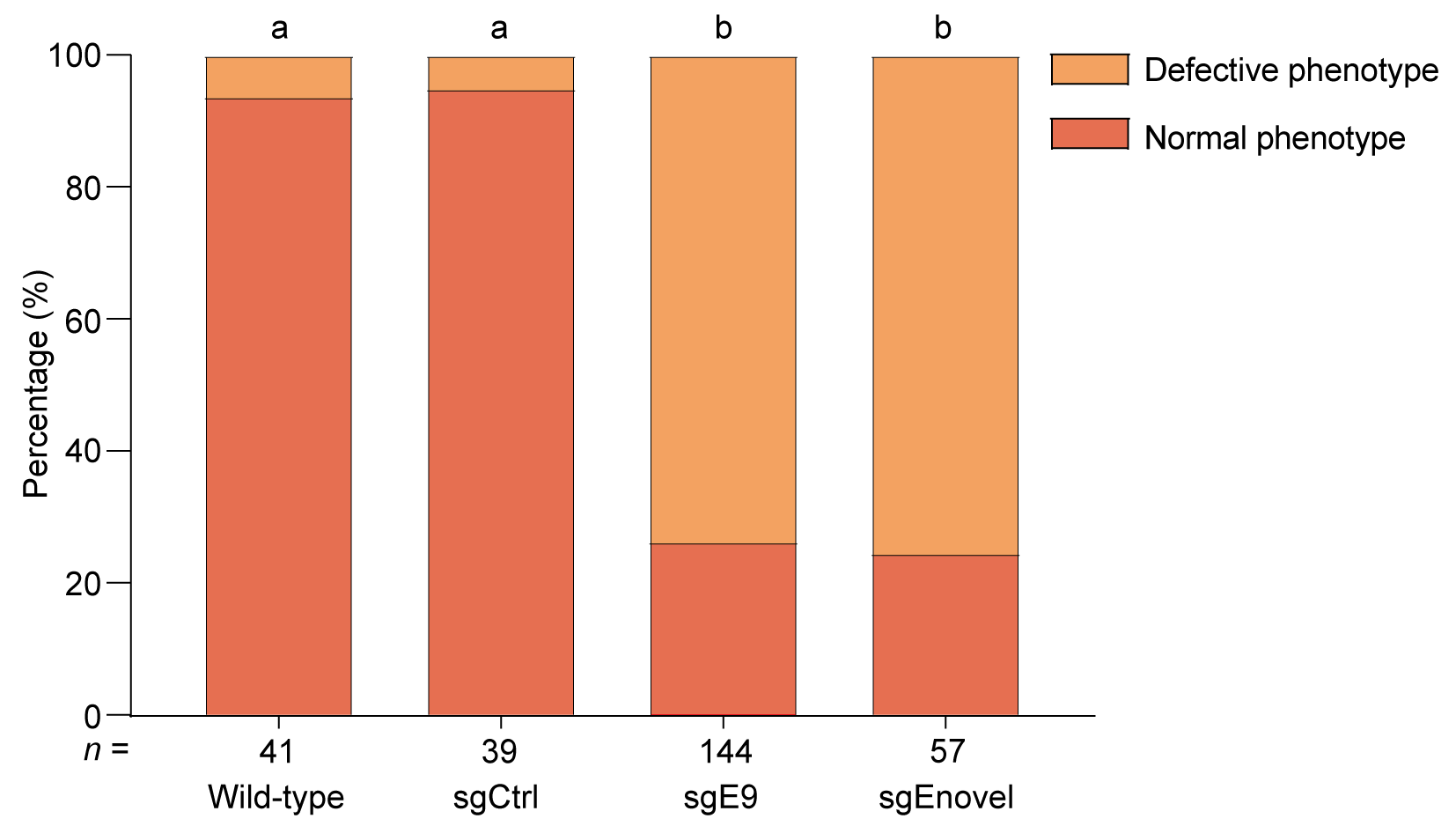


**Fig. S8 | CRISPRi targeting of *meis1b* enhancers impairs erythrocyte formation in zebrafish embryos.** Quantification of erythrocyte formation phenotypes, based on o-dianisidine staining of the yolk sac at 48 hpf (shown in Fig. 5F), in embryos injected with sgRNAs targeting the E9 and Enovel enhancer regions of the *meis1b* gene, alongside wild-type controls. Data represent the mean from all embryos (*n*) across three independent injections. Statistical comparisons were performed using the chi-square test followed by Bonferroni’s post-hoc correction; different letters indicate statistically significant differences (adjusted *P* < 0.0083). sgCtrl, scrambled non-targeting control sgRNA.

**Table S1 | Primer sequences used for sgRNA synthesis and quantitative RT-PCR assays in this study.**

| **sgRNA Primers for CRISPRi** | **Sequence (5'→ 3')** |
| --- | --- |
| sgTyr1 | GATCACTAATACGACTCACTATAGGCAGAGTGTAAAGCCTCTCGTTTTAGAGCTAGAAATAGC |
| sgTyr2 | GATCACTAATACGACTCACTATAGGGTGTGTGTAAAGCCTCTCGTTTTAGAGCTAGAAATAGC |
| sgTyr3 | GATCACTAATACGACTCACTATAGGAGGATACTGCGGCCCGTTGTTTTAGAGCTAGAAATAGC |
| sgTyr4 | GATCACTAATACGACTCACTATAGGTTGCGGCGAATGCAAGTTGTTTTAGAGCTAGAAATAGC |
| sgTS1 | GATCACTAATACGACTCACTATAGGATGCACAATCTGTCCCGGGTTTTAGAGCTAGAAATAGC |
| sgTS2 | GATCACTAATACGACTCACTATAGGTCAGCTGTTTGTCTGTGAGTTTTAGAGCTAGAAATAGC |
| sgTS3 | GATCACTAATACGACTCACTATAGGGTCCGTTTTAGAAGCCTGGTTTTAGAGCTAGAAATAGC |
| sgTS4 | GATCACTAATACGACTCACTATAAGCAGCTGACTTTTTTCCAGGTTTTAGAGCTAGAAATAGC |
| sgTS5 | GATCACTAATACGACTCACTATAGCTTGCTGTAACCTACCGAAGTTTTAGAGCTAGAAATAGC |
| sgTS6 | GATCACTAATACGACTCACTATATCGCCTCATCAAGACCTGCTGTTTTAGAGCTAGAAATAGC |
| sgZRS | GATCACTAATACGACTCACTATAGCTCCCGTACAAATGAGTGCGTTTTAGAGCTAGAAATAGC |
| sgsZRS | GATCACTAATACGACTCACTATAACACTTCTTAGAAGTCATGCGTTTTAGAGCTAGAAATAGC |
| sgNovel | GATCACTAATACGACTCACTATAGCTCTTCTAGGGATGATGGTGTTTTAGAGCTAGAAATAGC |
| sgE9 | GATCACTAATACGACTCACTATAAAATGGAGGTGTGCACAGCAGTTTTAGAGCTAGAAATAGC |
| sgEnovel | GATCACTAATACGACTCACTATAGCTACACAATACCAGGCAGGGTTTTAGAGCTAGAAATAGC |
| sgRps7-1 | GATCACTAATACGACTCACTATACTACTGTAAGACCTCGAGTCGTTTTAGAGCTAGAAATAGC |
| sgRps7-2 | GATCACTAATACGACTCACTATACTGCTACCAGATGAGCACGGGTTTTAGAGCTAGAAATAGC |
| sgRps7-3 | GATCACTAATACGACTCACTATACCACTTCCAGAGCGATCCGTGTTTTAGAGCTAGAAATAGC |
| sgPou3f1 | GATCACTAATACGACTCACTATACACTCCGACAGTCTTTCAGGGTTTTAGAGCTAGAAATAGC |
| sgSp8b | GATCACTAATACGACTCACTATAGGATCCGTCCGTCCTCCCAGGTTTTAGAGCTAGAAATAGC |
| sgCtrl (scrambled sgRNA) | GATCACTAATACGACTCACTATACCCCAGAAGTCCTCCAGTCCGTTTTAGAGCTAGAAATAGC |
| sgRNA Scaffold | AAAAGCACCGACTCGGTGCCACTTTTTCAAGTTGATAACGGACTAGCCTTATTTTAACTTGCTATTTCTAGCTCTAAAAC |
| **sgRNA Primers for CRISPR** | **Sequence (5'→ 3')** |
| sgTyr | GATCACTAATACGACTCACTATAGGGTGTGTGTGAAGCGTCTCGTTTTAGAGCTAGAAATAGC |
| **Primers for qRT-PCR** | **Sequence (5'→ 3')** |
| RT-tyr-F | TTCACCATCCCGTACTG |
| RT-tyr-R | GAGAACAGATCACCTTCCAGG |
| RT-α_e1_-F | CCAGGATGTTGATTGTCTAC |
| RT-α_e1_-R | CAGTCTTGCCGTGTTTC |
| RT-β_e1_-F | CTTGACCATCGTTGTTG |
| RT-β_e1_-R | GATGAATTTCTGGAAAGC |
| RT-α_e3_-F | CCTAAGCCCCAACTCTC |
| RT-α_e3_-R | CTCCCTTCAGGTCATCC |
| RT-β-actin-F | CGAGCTGTCTTCCCATCCA |
| RT-β-actin-R | TCACCAACGTAGCTGTCTTTCTG |
| RT-shha-F1 | AGACAAGAGCAAATACGGGACA |
| RT-shha-R1 | AGCCGAACCTGGGAAACA |
| RT-meis1b-F1 | AGGAGATGATGACGACCCTGAC |
| RT-meis1b-R1 | CTTACTGCTCGGTTGGATTGG |
| RT-rps7-F1 | AAAATAGTGAAGCCGAATGGC |
| RT-rps7-R1 | CAGGCACAAAGATGATGATGG |
| RT-pou3f1-F1 | CTGCCAACCTCCAACACC |
| RT-pou3f1-R1 | TGTAGCCCGTCTGCGAGT |
| RT-sp8b-F1 | CTCGGAATGGTGGTGGTTTA |
| RT-sp8b-R1 | GGTTGGTGGCTTGGCTCTT |
| **Primers for genotyping** | **Sequence (5'→ 3')** |
| GT-tyr-F | CTCTCCAGCAGTGTGTAAAGC |
| GT-tyr-F | ACAGAACCCTCGACCTGACT |

**Table S2 | Summary of CRISPR components injection concentrations and related parameters in different studies.**

| **Cas9 Type** | **gRNA concentration** | **Cas9**  **concentration** | **Application** | **gRNA:Cas9** | **Inject Amount** | **References** |
| --- | --- | --- | --- | --- | --- | --- |
| dCas9 mRNA  cas9 mRNA | 25 ng/μL | 100 ng/μL | Target *chd*, *oep* | 1:4 | 25 pg gRNA; 100 pg dCas9 | Tanaka et al., 2018 |
| dCas9 plasmid | 150 ng/μL | 300 ng/μL | Target *sox10*, *mitfa*, *mitfb*,*tyr* | 1:2 | 150 pg gRNA; 300 pg dCas9 | Barrientos et al., 2024 |
| Cas9 protein | 100 ng/μL | 800 ng/μL | Target *tyr*, *gol,* *slc24a5* | 1:8 | 100 pg gRNA; 800 pg Cas9 | Hu et al., 2017 |
| Cas9 mRNA | 100 ng/μL | 400 ng/μL |  | 1:4 | 100 pg gRNA; 400 pg Cas9 |  |
| Cas9 mRNA | 150 ng/μL | 1000 ng/μL | Target *hif3α* | 1:6.67 | 150 pg gRNA; 100 pg Cas9 | Cai et al., 2020 |
| Cas9 protein | 103.8 ng/μL | 361.2 ng/μL | Target *foxd3*, *tfap2a*, *rx3*, *bmp7* | 1:3.48 | 103 pg gRNA; 361.2 pg Cas9 | Hoshijima et al., 2019 |
| dCas9 mRNA | 120 ng/μL | 500 ng/μL | Induce the repressive histone modifcation | 1:4.16 | 120 pg gRNA; 500 pg dCas9 | Fukushima et al., 2019 |
|  |  | 750 ng/μL |  | 1:6.25 | 120 pg gRNA; 750 pg dCas9 |  |
|  |  | 710 ng/μL |  | 1:5.91 | 120 pg gRNA; 710 pg dCas9 |  |
| nls-zCas9-nls mRNA | 50 ng/μL | 150 ng/μL | Target *gol* | 1:3 | 50 pg gRNA; 150 pg Cas9 | Jao et al., 2013 |
|  | 150 ng/μL |  | Target *ddx19* | 1:1 | 150 pg gRNA; 150 pg Cas9 |  |
|  | 30 ng/μL |  | Target *tyr* | 1:5 | 30 pg gRNA; 150 pg Cas9 |  |
| dCas9 mRNA | 100 ng/μL | 250 ng/μL | Target *znfl1*, *pou5f3*, *sall4* | 1:2.5 | 100 pg gRNA; 250 pg dCas9 | Dong et al., 2017 |
| dCas9 mRNA | 100 ng/μL | 250 ng/μL | Target *scn1laa* | 1:2.5 | 50 pg gRNA; 125 pg dCas9 | Weuring et al., 2021 |

**Table S3 | Enhancer-Promoter loops identified in this study.**

|  | Chr | Promoter | | Enhancer | | Dist (kb) | target_gene_ID |
| --- | --- | --- | --- | --- | --- | --- | --- |
|  |  | start_1 | end_1 | start_2 | end_2 |  |  |
| 1 | 1 | 2300000 | 2325000 | 2400000 | 2425000 | 100 | ENSDARG00000040245 |
| 2 | 1 | 2400000 | 2425000 | 2300000 | 2325000 | 100 | ENSDARG00000074381 |
| 3 | 1 | 7550000 | 7575000 | 7650000 | 7675000 | 100 | ENSDARG00000017441 |
| 4 | 1 | 7650000 | 7675000 | 7550000 | 7575000 | 100 | ENSDARG00000018319 |
| 5 | 1 | 8700000 | 8725000 | 8900000 | 8925000 | 200 | ENSDARG00000012458 |
| 6 | 1 | 13475000 | 13500000 | 17950000 | 17975000 | 4475 | ENSDARG00000117726 |
| 7 | 1 | 23555000 | 23560000 | 22230000 | 22235000 | 1325 | ENSDARG00000055099 |
| 8 | 1 | 26650000 | 26675000 | 26050000 | 26075000 | 600 | ENSDARG00000023330 |
| 9 | 1 | 32075000 | 32100000 | 26225000 | 26250000 | 5850 | ENSDARG00000053241 |
| 10 | 1 | 36200000 | 36225000 | 35950000 | 35975000 | 250 | ENSDARG00000036995 |
| 11 | 1 | 35950000 | 35975000 | 36200000 | 36225000 | 250 | ENSDARG00000060383 |
| 12 | 1 | 37300000 | 37325000 | 37175000 | 37200000 | 125 | ENSDARG00000034093 |
| 13 | 1 | 38650000 | 38660000 | 38750000 | 38760000 | 100 | ENSDARG00000004621 |
| 14 | 1 | 45550000 | 45575000 | 45350000 | 45375000 | 200 | ENSDARG00000030376 |
| 15 | 1 | 50600000 | 50625000 | 49525000 | 49550000 | 1075 | ENSDARG00000010655 |
| 16 | 1 | 49700000 | 49725000 | 49825000 | 49850000 | 125 | ENSDARG00000094063 |
| 17 | 1 | 50850000 | 50875000 | 50950000 | 50975000 | 100 | ENSDARG00000075043 |
| 18 | 1 | 51470000 | 51480000 | 51420000 | 51430000 | 50 | ENSDARG00000052435 |
| 19 | 1 | 53750000 | 53775000 | 53875000 | 53900000 | 125 | ENSDARG00000087205 |
| 20 | 2 | 2200000 | 2225000 | 2325000 | 2350000 | 125 | ENSDARG00000063451 |
| 21 | 2 | 2925000 | 2950000 | 2625000 | 2650000 | 300 | ENSDARG00000101799 |
| 22 | 2 | 5925000 | 5950000 | 4450000 | 4475000 | 1475 | ENSDARG00000075980 |
| 23 | 2 | 10925000 | 10950000 | 11025000 | 11050000 | 100 | ENSDARG00000058237 |
| 24 | 2 | 14375000 | 14400000 | 13150000 | 13175000 | 1225 | ENSDARG00000101215 |
| 25 | 2 | 13775000 | 13800000 | 16725000 | 16750000 | 2950 | ENSDARG00000095477 |
| 26 | 2 | 14030000 | 14040000 | 14670000 | 14680000 | 640 | ENSDARG00000097095 |
| 27 | 2 | 23075000 | 23100000 | 23300000 | 23325000 | 225 | ENSDARG00000005458 |
| 28 | 2 | 32725000 | 32750000 | 32575000 | 32600000 | 150 | ENSDARG00000032951 |
| 29 | 2 | 35725000 | 35750000 | 35100000 | 35125000 | 625 | ENSDARG00000011326 |
| 30 | 2 | 35725000 | 35750000 | 35100000 | 35125000 | 625 | ENSDARG00000095663 |
| 31 | 2 | 35600000 | 35625000 | 35700000 | 35725000 | 100 | ENSDARG00000014731 |
| 32 | 2 | 36940000 | 36950000 | 37020000 | 37030000 | 80 | ENSDARG00000013576 |
| 33 | 2 | 41600000 | 41625000 | 41700000 | 41725000 | 100 | ENSDARG00000056490 |
| 34 | 2 | 41600000 | 41625000 | 41800000 | 41825000 | 200 | ENSDARG00000056490 |
| 35 | 2 | 41720000 | 41730000 | 41810000 | 41820000 | 90 | ENSDARG00000056490 |
| 36 | 2 | 42050000 | 42075000 | 42225000 | 42250000 | 175 | ENSDARG00000091628 |
| 37 | 2 | 43725000 | 43750000 | 42225000 | 42250000 | 1500 | ENSDARG00000074131 |
| 38 | 2 | 42700000 | 42725000 | 42300000 | 42325000 | 400 | ENSDARG00000017004 |
| 39 | 2 | 42700000 | 42725000 | 42300000 | 42325000 | 400 | ENSDARG00000053366 |
| 40 | 2 | 42300000 | 42325000 | 42700000 | 42725000 | 400 | ENSDARG00000053450 |
| 41 | 2 | 42300000 | 42325000 | 43125000 | 43150000 | 825 | ENSDARG00000053450 |
| 42 | 2 | 42700000 | 42725000 | 43125000 | 43150000 | 425 | ENSDARG00000017004 |
| 43 | 2 | 42700000 | 42725000 | 43125000 | 43150000 | 425 | ENSDARG00000053366 |
| 44 | 2 | 42700000 | 42725000 | 43725000 | 43750000 | 1025 | ENSDARG00000017004 |
| 45 | 2 | 42700000 | 42725000 | 43725000 | 43750000 | 1025 | ENSDARG00000053366 |
| 46 | 2 | 43725000 | 43750000 | 42700000 | 42725000 | 1025 | ENSDARG00000074131 |
| 47 | 2 | 57925000 | 57950000 | 48800000 | 48825000 | 9125 | ENSDARG00000079930 |
| 48 | 2 | 52525000 | 52550000 | 58100000 | 58125000 | 5575 | ENSDARG00000053326 |
| 49 | 2 | 58275000 | 58300000 | 58150000 | 58175000 | 125 | ENSDARG00000117348 |
| 50 | 3 | 17500000 | 17525000 | 17375000 | 17400000 | 125 | ENSDARG00000062575 |
| 51 | 3 | 21280000 | 21290000 | 21340000 | 21350000 | 60 | ENSDARG00000075249 |
| 52 | 3 | 23375000 | 23400000 | 22850000 | 22875000 | 525 | ENSDARG00000107397 |
| 53 | 3 | 23350000 | 23360000 | 23400000 | 23410000 | 50 | ENSDARG00000079912 |
| 54 | 3 | 23700000 | 23725000 | 23925000 | 23950000 | 225 | ENSDARG00000013057 |
| 55 | 3 | 28600000 | 28625000 | 27950000 | 27975000 | 650 | ENSDARG00000019191 |
| 56 | 3 | 29670000 | 29680000 | 29850000 | 29860000 | 180 | ENSDARG00000073718 |
| 57 | 3 | 34325000 | 34350000 | 34425000 | 34450000 | 100 | ENSDARG00000076847 |
| 58 | 3 | 40520000 | 40530000 | 40350000 | 40360000 | 170 | ENSDARG00000037870 |
| 59 | 3 | 40350000 | 40360000 | 40520000 | 40530000 | 170 | ENSDARG00000059690 |
| 60 | 3 | 40350000 | 40360000 | 40630000 | 40640000 | 280 | ENSDARG00000059690 |
| 61 | 3 | 40350000 | 40375000 | 42325000 | 42350000 | 1975 | ENSDARG00000059690 |
| 62 | 3 | 40525000 | 40550000 | 40625000 | 40650000 | 100 | ENSDARG00000037870 |
| 63 | 3 | 42030000 | 42040000 | 42080000 | 42090000 | 50 | ENSDARG00000034473 |
| 64 | 3 | 43350000 | 43375000 | 43125000 | 43150000 | 225 | ENSDARG00000102976 |
| 65 | 3 | 55110000 | 55135000 | 55055000 | 55080000 | 55 | ENSDARG00000089124 |
| 66 | 3 | 55140000 | 55150000 | 55062500 | 55072500 | 77.5 | ENSDARG00000079305 |
| 67 | 3 | 58675000 | 58700000 | 55350000 | 55375000 | 3325 | ENSDARG00000069839 |
| 68 | 3 | 60710000 | 60715000 | 60675000 | 60680000 | 35 | ENSDARG00000115335 |
| 69 | 3 | 61100000 | 61125000 | 60975000 | 61000000 | 125 | ENSDARG00000018903 |
| 70 | 4 | 2925000 | 2950000 | 2750000 | 2775000 | 175 | ENSDARG00000006038 |
| 71 | 4 | 5300000 | 5325000 | 4950000 | 4975000 | 350 | ENSDARG00000063481 |
| 72 | 4 | 9975000 | 10000000 | 9875000 | 9900000 | 100 | ENSDARG00000062968 |
| 73 | 4 | 12775000 | 12800000 | 12875000 | 12900000 | 100 | ENSDARG00000053136 |
| 74 | 4 | 15100000 | 15110000 | 15050000 | 15060000 | 50 | ENSDARG00000000018 |
| 75 | 4 | 16300000 | 16325000 | 16050000 | 16075000 | 250 | ENSDARG00000056954 |
| 76 | 4 | 16540000 | 16550000 | 16450000 | 16460000 | 90 | ENSDARG00000027249 |
| 77 | 4 | 17950000 | 17975000 | 17850000 | 17875000 | 100 | ENSDARG00000003512 |
| 78 | 4 | 18100000 | 18125000 | 18200000 | 18225000 | 100 | ENSDARG00000003512 |
| 79 | 4 | 22300000 | 22325000 | 22150000 | 22175000 | 150 | ENSDARG00000055360 |
| 80 | 4 | 25850000 | 25875000 | 26000000 | 26025000 | 150 | ENSDARG00000045527 |
| 81 | 4 | 26025000 | 26050000 | 25925000 | 25950000 | 100 | ENSDARG00000089861 |
| 82 | 4 | 26050000 | 26075000 | 28875000 | 28900000 | 2825 | ENSDARG00000089908 |
| 83 | 4 | 74125000 | 74150000 | 74275000 | 74300000 | 150 | ENSDARG00000105760 |
| 84 | 4 | 74125000 | 74150000 | 74950000 | 74975000 | 825 | ENSDARG00000105760 |
| 85 | 4 | 77560000 | 77570000 | 76390000 | 76400000 | 1170 | ENSDARG00000081218 |
| 86 | 4 | 76475000 | 76500000 | 77000000 | 77025000 | 525 | ENSDARG00000029105 |
| 87 | 5 | 17025000 | 17050000 | 16725000 | 16750000 | 300 | ENSDARG00000095235 |
| 88 | 5 | 19325000 | 19350000 | 19100000 | 19125000 | 225 | ENSDARG00000092739 |
| 89 | 5 | 20200000 | 20225000 | 20375000 | 20400000 | 175 | ENSDARG00000035601 |
| 90 | 5 | 30175000 | 30200000 | 30325000 | 30350000 | 150 | ENSDARG00000007709 |
| 91 | 5 | 34825000 | 34850000 | 34625000 | 34650000 | 200 | ENSDARG00000108823 |
| 92 | 5 | 34975000 | 35000000 | 35150000 | 35175000 | 175 | ENSDARG00000035400 |
| 93 | 5 | 36400000 | 36425000 | 36000000 | 36025000 | 400 | ENSDARG00000060152 |
| 94 | 5 | 36320000 | 36330000 | 36030000 | 36040000 | 290 | ENSDARG00000007723 |
| 95 | 5 | 36850000 | 36875000 | 37075000 | 37100000 | 225 | ENSDARG00000035326 |
| 96 | 5 | 41700000 | 41725000 | 47525000 | 47550000 | 5825 | ENSDARG00000052897 |
| 97 | 5 | 44925000 | 44950000 | 44825000 | 44850000 | 100 | ENSDARG00000060102 |
| 98 | 5 | 48170000 | 48180000 | 48010000 | 48020000 | 160 | ENSDARG00000081362 |
| 99 | 5 | 55750000 | 55775000 | 56450000 | 56475000 | 700 | ENSDARG00000067656 |
| 100 | 5 | 58990000 | 59000000 | 58840000 | 58850000 | 150 | ENSDARG00000026454 |
| 101 | 5 | 63825000 | 63850000 | 60650000 | 60675000 | 3175 | ENSDARG00000035132 |
| 102 | 5 | 61820000 | 61830000 | 69680000 | 69690000 | 7860 | ENSDARG00000079780 |
| 103 | 5 | 69860000 | 69870000 | 69920000 | 69930000 | 60 | ENSDARG00000011537 |
| 104 | 5 | 71750000 | 71775000 | 71900000 | 71925000 | 150 | ENSDARG00000054874 |
| 105 | 6 | 13725000 | 13750000 | 13225000 | 13250000 | 500 | ENSDARG00000038283 |
| 106 | 6 | 15875000 | 15900000 | 15650000 | 15675000 | 225 | ENSDARG00000057276 |
| 107 | 6 | 15875000 | 15900000 | 15650000 | 15675000 | 225 | ENSDARG00000097207 |
| 108 | 6 | 17790000 | 17800000 | 23600000 | 23610000 | 5810 | ENSDARG00000114314 |
| 109 | 6 | 23810000 | 23820000 | 23870000 | 23880000 | 60 | ENSDARG00000100003 |
| 110 | 6 | 24675000 | 24700000 | 25075000 | 25100000 | 400 | ENSDARG00000102025 |
| 111 | 6 | 24800000 | 24825000 | 24900000 | 24925000 | 100 | ENSDARG00000104361 |
| 112 | 6 | 29045000 | 29050000 | 29080000 | 29085000 | 35 | ENSDARG00000070839 |
| 113 | 6 | 31775000 | 31800000 | 31975000 | 32000000 | 200 | ENSDARG00000103867 |
| 114 | 6 | 46450000 | 46475000 | 37375000 | 37400000 | 9075 | ENSDARG00000028862 |
| 115 | 6 | 46875000 | 46900000 | 47550000 | 47575000 | 675 | ENSDARG00000097613 |
| 116 | 6 | 52350000 | 52375000 | 52725000 | 52750000 | 375 | ENSDARG00000096319 |
| 117 | 7 | 1425000 | 1450000 | 875000 | 900000 | 550 | ENSDARG00000105485 |
| 118 | 7 | 8600000 | 8625000 | 8875000 | 8900000 | 275 | ENSDARG00000098977 |
| 119 | 7 | 18150000 | 18175000 | 20825000 | 20850000 | 2675 | ENSDARG00000087452 |
| 120 | 7 | 24375000 | 24400000 | 26775000 | 26800000 | 2400 | ENSDARG00000087017 |
| 121 | 7 | 24875000 | 24900000 | 24975000 | 25000000 | 100 | ENSDARG00000040224 |
| 122 | 7 | 27330000 | 27340000 | 27420000 | 27430000 | 90 | ENSDARG00000015536 |
| 123 | 7 | 29000000 | 29025000 | 28850000 | 28875000 | 150 | ENSDARG00000012030 |
| 124 | 7 | 29000000 | 29025000 | 28850000 | 28875000 | 150 | ENSDARG00000061149 |
| 125 | 7 | 31600000 | 31625000 | 31475000 | 31500000 | 125 | ENSDARG00000075158 |
| 126 | 7 | 31650000 | 31675000 | 31750000 | 31775000 | 100 | ENSDARG00000076919 |
| 127 | 7 | 31900000 | 31925000 | 32050000 | 32075000 | 150 | ENSDARG00000027322 |
| 128 | 7 | 31925000 | 31950000 | 32225000 | 32250000 | 300 | ENSDARG00000018817 |
| 129 | 7 | 33475000 | 33500000 | 34050000 | 34075000 | 575 | ENSDARG00000052688 |
| 130 | 7 | 35075000 | 35100000 | 35175000 | 35200000 | 100 | ENSDARG00000069089 |
| 131 | 7 | 39750000 | 39775000 | 39925000 | 39950000 | 175 | ENSDARG00000010021 |
| 132 | 7 | 39750000 | 39775000 | 40025000 | 40050000 | 275 | ENSDARG00000010021 |
| 133 | 7 | 40880000 | 40890000 | 40667000 | 40677000 | 213 | ENSDARG00000105553 |
| 134 | 7 | 40880000 | 40890000 | 40810000 | 40820000 | 70 | ENSDARG00000105553 |
| 135 | 7 | 44700000 | 44710000 | 44630000 | 44640000 | 70 | ENSDARG00000035924 |
| 136 | 7 | 53010000 | 53020000 | 47760000 | 47770000 | 5250 | ENSDARG00000104600 |
| 137 | 7 | 48660000 | 48670000 | 48600000 | 48610000 | 60 | ENSDARG00000010878 |
| 138 | 7 | 51525000 | 51550000 | 49100000 | 49125000 | 2425 | ENSDARG00000089066 |
| 139 | 7 | 52550000 | 52575000 | 52700000 | 52725000 | 150 | ENSDARG00000004714 |
| 140 | 7 | 52550000 | 52575000 | 52700000 | 52725000 | 150 | ENSDARG00000098896 |
| 141 | 7 | 54670000 | 54680000 | 54790000 | 54800000 | 120 | ENSDARG00000101637 |
| 142 | 7 | 54670000 | 54680000 | 54960000 | 54970000 | 290 | ENSDARG00000101637 |
| 143 | 7 | 55525000 | 55550000 | 55925000 | 55950000 | 400 | ENSDARG00000003519 |
| 144 | 7 | 62300000 | 62325000 | 62000000 | 62025000 | 300 | ENSDARG00000013842 |
| 145 | 7 | 62000000 | 62025000 | 62300000 | 62325000 | 300 | ENSDARG00000042728 |
| 146 | 7 | 65925000 | 65950000 | 66025000 | 66050000 | 100 | ENSDARG00000059483 |
| 147 | 7 | 66025000 | 66050000 | 65925000 | 65950000 | 100 | ENSDARG00000100567 |
| 148 | 7 | 65925000 | 65950000 | 66025000 | 66050000 | 100 | ENSDARG00000112859 |
| 149 | 8 | 4025000 | 4050000 | 4325000 | 4350000 | 300 | ENSDARG00000098201 |
| 150 | 8 | 23450000 | 23475000 | 13275000 | 13300000 | 10175 | ENSDARG00000078279 |
| 151 | 8 | 17900000 | 17925000 | 17775000 | 17800000 | 125 | ENSDARG00000057419 |
| 152 | 8 | 19400000 | 19410000 | 19460000 | 19470000 | 60 | ENSDARG00000098592 |
| 153 | 8 | 22610000 | 22620000 | 22560000 | 22570000 | 50 | ENSDARG00000098823 |
| 154 | 8 | 32775000 | 32800000 | 24275000 | 24300000 | 8500 | ENSDARG00000092658 |
| 155 | 8 | 40275000 | 40300000 | 40425000 | 40450000 | 150 | ENSDARG00000011515 |
| 156 | 8 | 40275000 | 40300000 | 40425000 | 40450000 | 150 | ENSDARG00000036593 |
| 157 | 8 | 45350000 | 45375000 | 46025000 | 46050000 | 675 | ENSDARG00000002597 |
| 158 | 8 | 49150000 | 49175000 | 49425000 | 49450000 | 275 | ENSDARG00000022684 |
| 159 | 9 | 3150000 | 3175000 | 3750000 | 3775000 | 600 | ENSDARG00000011652 |
| 160 | 9 | 5350000 | 5375000 | 5650000 | 5675000 | 300 | ENSDARG00000059209 |
| 161 | 9 | 8450000 | 8475000 | 8025000 | 8050000 | 425 | ENSDARG00000075282 |
| 162 | 9 | 8975000 | 9000000 | 9100000 | 9125000 | 125 | ENSDARG00000019116 |
| 163 | 9 | 12475000 | 12500000 | 12575000 | 12600000 | 100 | ENSDARG00000003421 |
| 164 | 9 | 12950000 | 12975000 | 13075000 | 13100000 | 125 | ENSDARG00000020834 |
| 165 | 9 | 20525000 | 20550000 | 20850000 | 20875000 | 325 | ENSDARG00000078643 |
| 166 | 9 | 23150000 | 23175000 | 23050000 | 23075000 | 100 | ENSDARG00000077643 |
| 167 | 9 | 23355000 | 23360000 | 23320000 | 23325000 | 35 | ENSDARG00000032708 |
| 168 | 9 | 25975000 | 26000000 | 25525000 | 25550000 | 450 | ENSDARG00000062349 |
| 169 | 9 | 31550000 | 31575000 | 31125000 | 31150000 | 425 | ENSDARG00000056191 |
| 170 | 9 | 34500000 | 34525000 | 34850000 | 34875000 | 350 | ENSDARG00000076004 |
| 171 | 9 | 36950000 | 36975000 | 37125000 | 37150000 | 175 | ENSDARG00000074079 |
| 172 | 9 | 42150000 | 42175000 | 39425000 | 39450000 | 2725 | ENSDARG00000078415 |
| 173 | 10 | 1130000 | 1140000 | 1650000 | 1660000 | 520 | ENSDARG00000102742 |
| 174 | 10 | 4075000 | 4100000 | 4200000 | 4225000 | 125 | ENSDARG00000036005 |
| 175 | 10 | 4075000 | 4100000 | 4450000 | 4475000 | 375 | ENSDARG00000036005 |
| 176 | 10 | 4300000 | 4325000 | 11850000 | 11875000 | 7550 | ENSDARG00000075802 |
| 177 | 10 | 10800000 | 10825000 | 6200000 | 6225000 | 4600 | ENSDARG00000004296 |
| 178 | 10 | 10725000 | 10750000 | 10400000 | 10425000 | 325 | ENSDARG00000078492 |
| 179 | 10 | 10860000 | 10865000 | 10905000 | 10910000 | 45 | ENSDARG00000091419 |
| 180 | 10 | 23025000 | 23050000 | 13975000 | 14000000 | 9050 | ENSDARG00000086665 |
| 181 | 10 | 15960000 | 15970000 | 15880000 | 15890000 | 80 | ENSDARG00000080923 |
| 182 | 10 | 19600000 | 19625000 | 20050000 | 20075000 | 450 | ENSDARG00000117243 |
| 183 | 10 | 21650000 | 21675000 | 21850000 | 21875000 | 200 | ENSDARG00000097170 |
| 184 | 10 | 21650000 | 21675000 | 21850000 | 21875000 | 200 | ENSDARG00000103602 |
| 185 | 10 | 21650000 | 21675000 | 21850000 | 21875000 | 200 | ENSDARG00000104598 |
| 186 | 10 | 22475000 | 22500000 | 22300000 | 22325000 | 175 | ENSDARG00000079251 |
| 187 | 10 | 22475000 | 22500000 | 22300000 | 22325000 | 175 | ENSDARG00000117144 |
| 188 | 10 | 22525000 | 22550000 | 22675000 | 22700000 | 150 | ENSDARG00000078691 |
| 189 | 10 | 25350000 | 25375000 | 25700000 | 25725000 | 350 | ENSDARG00000069143 |
| 190 | 10 | 25700000 | 25725000 | 25350000 | 25375000 | 350 | ENSDARG00000077093 |
| 191 | 10 | 28060000 | 28070000 | 28110000 | 28120000 | 50 | ENSDARG00000053884 |
| 192 | 10 | 32900000 | 32925000 | 33150000 | 33175000 | 250 | ENSDARG00000078459 |
| 193 | 10 | 33080000 | 33090000 | 33150000 | 33160000 | 70 | ENSDARG00000078459 |
| 194 | 10 | 35050000 | 35060000 | 35110000 | 35120000 | 60 | ENSDARG00000078658 |
| 195 | 10 | 35240000 | 35250000 | 35190000 | 35200000 | 50 | ENSDARG00000061378 |
| 196 | 10 | 36575000 | 36600000 | 36025000 | 36050000 | 550 | ENSDARG00000043148 |
| 197 | 10 | 36375000 | 36400000 | 36050000 | 36075000 | 325 | ENSDARG00000103268 |
| 198 | 10 | 38240000 | 38250000 | 38380000 | 38390000 | 140 | ENSDARG00000012314 |
| 199 | 10 | 39130000 | 39135000 | 39245000 | 39250000 | 115 | ENSDARG00000041811 |
| 200 | 10 | 42350000 | 42375000 | 42175000 | 42200000 | 175 | ENSDARG00000008170 |
| 201 | 11 | 3850000 | 3875000 | 3650000 | 3675000 | 200 | ENSDARG00000059327 |
| 202 | 11 | 8650000 | 8675000 | 8350000 | 8375000 | 300 | ENSDARG00000058696 |
| 203 | 11 | 10650000 | 10675000 | 10800000 | 10825000 | 150 | ENSDARG00000079742 |
| 204 | 11 | 18700000 | 18710000 | 18780000 | 18790000 | 80 | ENSDARG00000040764 |
| 205 | 11 | 22075000 | 22100000 | 21600000 | 21625000 | 475 | ENSDARG00000076120 |
| 206 | 11 | 23610000 | 23620000 | 23690000 | 23700000 | 80 | ENSDARG00000086927 |
| 207 | 11 | 25150000 | 25175000 | 24625000 | 24650000 | 525 | ENSDARG00000099887 |
| 208 | 11 | 25475000 | 25500000 | 33950000 | 33975000 | 8475 | ENSDARG00000044861 |
| 209 | 11 | 33950000 | 33975000 | 25475000 | 25500000 | 8475 | ENSDARG00000113099 |
| 210 | 11 | 27500000 | 27525000 | 27625000 | 27650000 | 125 | ENSDARG00000105504 |
| 211 | 11 | 30625000 | 30650000 | 30525000 | 30550000 | 100 | ENSDARG00000070401 |
| 212 | 11 | 33175000 | 33200000 | 32750000 | 32775000 | 425 | ENSDARG00000117268 |
| 213 | 11 | 33900000 | 33925000 | 34475000 | 34500000 | 575 | ENSDARG00000030687 |
| 214 | 11 | 35050000 | 35060000 | 35160000 | 35170000 | 110 | ENSDARG00000070360 |
| 215 | 11 | 40650000 | 40675000 | 40825000 | 40850000 | 175 | ENSDARG00000004302 |
| 216 | 11 | 43025000 | 43050000 | 43175000 | 43200000 | 150 | ENSDARG00000112439 |
| 217 | 12 | 2480000 | 2490000 | 2400000 | 2410000 | 80 | ENSDARG00000096550 |
| 218 | 12 | 3075000 | 3100000 | 3525000 | 3550000 | 450 | ENSDARG00000019746 |
| 219 | 12 | 15600000 | 15625000 | 17275000 | 17300000 | 1675 | ENSDARG00000027070 |
| 220 | 12 | 15600000 | 15625000 | 23550000 | 23575000 | 7950 | ENSDARG00000027070 |
| 221 | 12 | 16280000 | 16290000 | 16340000 | 16350000 | 60 | ENSDARG00000014554 |
| 222 | 12 | 25600000 | 25625000 | 25400000 | 25425000 | 200 | ENSDARG00000054878 |
| 223 | 12 | 25400000 | 25425000 | 25600000 | 25625000 | 200 | ENSDARG00000096769 |
| 224 | 12 | 28350000 | 28375000 | 28475000 | 28500000 | 125 | ENSDARG00000005134 |
| 225 | 12 | 28350000 | 28375000 | 28475000 | 28500000 | 125 | ENSDARG00000045087 |
| 226 | 12 | 31775000 | 31800000 | 32050000 | 32075000 | 275 | ENSDARG00000071426 |
| 227 | 12 | 33450000 | 33475000 | 33550000 | 33575000 | 100 | ENSDARG00000024184 |
| 228 | 12 | 33550000 | 33575000 | 33450000 | 33475000 | 100 | ENSDARG00000060868 |
| 229 | 12 | 33450000 | 33475000 | 33550000 | 33575000 | 100 | ENSDARG00000087657 |
| 230 | 12 | 46575000 | 46600000 | 46100000 | 46125000 | 475 | ENSDARG00000076079 |
| 231 | 13 | 5560000 | 5570000 | 5490000 | 5500000 | 70 | ENSDARG00000012078 |
| 232 | 13 | 29400000 | 29425000 | 27825000 | 27850000 | 1575 | ENSDARG00000015854 |
| 233 | 13 | 31600000 | 31625000 | 31700000 | 31725000 | 100 | ENSDARG00000039304 |
| 234 | 13 | 49425000 | 49450000 | 49325000 | 49350000 | 100 | ENSDARG00000004702 |
| 235 | 14 | 4210000 | 4220000 | 4270000 | 4280000 | 60 | ENSDARG00000089009 |
| 236 | 14 | 17410000 | 17420000 | 10490000 | 10500000 | 6920 | ENSDARG00000090319 |
| 237 | 14 | 11425000 | 11450000 | 11900000 | 11925000 | 475 | ENSDARG00000062788 |
| 238 | 14 | 17400000 | 17425000 | 13675000 | 13700000 | 3725 | ENSDARG00000090319 |
| 239 | 14 | 14830000 | 14840000 | 14740000 | 14750000 | 90 | ENSDARG00000100366 |
| 240 | 14 | 14830000 | 14840000 | 14740000 | 14750000 | 90 | ENSDARG00000102120 |
| 241 | 14 | 17400000 | 17425000 | 17850000 | 17875000 | 450 | ENSDARG00000090319 |
| 242 | 14 | 17400000 | 17425000 | 22975000 | 23000000 | 5575 | ENSDARG00000090319 |
| 243 | 14 | 24870000 | 24880000 | 24970000 | 24980000 | 100 | ENSDARG00000002463 |
| 244 | 14 | 27100000 | 27125000 | 33350000 | 33375000 | 6250 | ENSDARG00000105441 |
| 245 | 14 | 30910000 | 30920000 | 30800000 | 30810000 | 110 | ENSDARG00000055792 |
| 246 | 14 | 32250000 | 32275000 | 31900000 | 31925000 | 350 | ENSDARG00000035056 |
| 247 | 14 | 32250000 | 32260000 | 32130000 | 32140000 | 120 | ENSDARG00000035056 |
| 248 | 14 | 32250000 | 32275000 | 32425000 | 32450000 | 175 | ENSDARG00000035056 |
| 249 | 14 | 32530000 | 32540000 | 32630000 | 32640000 | 100 | ENSDARG00000036442 |
| 250 | 14 | 32625000 | 32650000 | 32725000 | 32750000 | 100 | ENSDARG00000036442 |
| 251 | 14 | 32625000 | 32650000 | 32800000 | 32825000 | 175 | ENSDARG00000036442 |
| 252 | 14 | 33075000 | 33100000 | 33175000 | 33200000 | 100 | ENSDARG00000076796 |
| 253 | 14 | 38825000 | 38850000 | 36850000 | 36875000 | 1975 | ENSDARG00000036162 |
| 254 | 14 | 36850000 | 36875000 | 38825000 | 38850000 | 1975 | ENSDARG00000089767 |
| 255 | 14 | 38825000 | 38850000 | 36850000 | 36875000 | 1975 | ENSDARG00000101935 |
| 256 | 14 | 38825000 | 38850000 | 36850000 | 36875000 | 1975 | ENSDARG00000103996 |
| 257 | 14 | 38925000 | 38950000 | 39175000 | 39200000 | 250 | ENSDARG00000036155 |
| 258 | 14 | 41250000 | 41275000 | 40850000 | 40875000 | 400 | ENSDARG00000052565 |
| 259 | 14 | 46000000 | 46025000 | 46100000 | 46125000 | 100 | ENSDARG00000059944 |
| 260 | 14 | 46100000 | 46125000 | 46000000 | 46025000 | 100 | ENSDARG00000071353 |
| 261 | 14 | 46100000 | 46125000 | 46000000 | 46025000 | 100 | ENSDARG00000103101 |
| 262 | 15 | 8645000 | 8650000 | 8760000 | 8765000 | 115 | ENSDARG00000062577 |
| 263 | 15 | 14350000 | 14375000 | 14250000 | 14275000 | 100 | ENSDARG00000099927 |
| 264 | 15 | 15500000 | 15525000 | 15750000 | 15775000 | 250 | ENSDARG00000045626 |
| 265 | 15 | 16175000 | 16200000 | 16300000 | 16325000 | 125 | ENSDARG00000030945 |
| 266 | 15 | 16300000 | 16325000 | 16175000 | 16200000 | 125 | ENSDARG00000033978 |
| 267 | 15 | 16175000 | 16200000 | 16300000 | 16325000 | 125 | ENSDARG00000042509 |
| 268 | 15 | 17650000 | 17675000 | 22275000 | 22300000 | 4625 | ENSDARG00000058252 |
| 269 | 15 | 18100000 | 18125000 | 17950000 | 17975000 | 150 | ENSDARG00000031214 |
| 270 | 15 | 21825000 | 21850000 | 21700000 | 21725000 | 125 | ENSDARG00000062315 |
| 271 | 15 | 21825000 | 21850000 | 21700000 | 21725000 | 125 | ENSDARG00000096853 |
| 272 | 15 | 22410000 | 22420000 | 22320000 | 22330000 | 90 | ENSDARG00000084849 |
| 273 | 15 | 25300000 | 25325000 | 25200000 | 25225000 | 100 | ENSDARG00000032013 |
| 274 | 15 | 28800000 | 28810000 | 28630000 | 28640000 | 170 | ENSDARG00000090386 |
| 275 | 15 | 28900000 | 28925000 | 29300000 | 29325000 | 400 | ENSDARG00000008808 |
| 276 | 15 | 31500000 | 31525000 | 31875000 | 31900000 | 375 | ENSDARG00000030479 |
| 277 | 15 | 31500000 | 31525000 | 31875000 | 31900000 | 375 | ENSDARG00000089689 |
| 278 | 16 | 6200000 | 6210000 | 6330000 | 6340000 | 130 | ENSDARG00000014571 |
| 279 | 16 | 6950000 | 6975000 | 7200000 | 7225000 | 250 | ENSDARG00000070795 |
| 280 | 16 | 12290000 | 12300000 | 7900000 | 7910000 | 4390 | ENSDARG00000117101 |
| 281 | 16 | 10850000 | 10875000 | 10950000 | 10975000 | 100 | ENSDARG00000002758 |
| 282 | 16 | 11240000 | 11250000 | 11180000 | 11190000 | 60 | ENSDARG00000077143 |
| 283 | 16 | 13660000 | 13670000 | 13610000 | 13620000 | 50 | ENSDARG00000062462 |
| 284 | 16 | 13800000 | 13825000 | 13700000 | 13725000 | 100 | ENSDARG00000062374 |
| 285 | 16 | 19525000 | 19550000 | 19375000 | 19400000 | 150 | ENSDARG00000056666 |
| 286 | 16 | 20850000 | 20875000 | 19625000 | 19650000 | 1225 | ENSDARG00000056856 |
| 287 | 16 | 23700000 | 23725000 | 19750000 | 19775000 | 3950 | ENSDARG00000019753 |
| 288 | 16 | 20500000 | 20525000 | 20700000 | 20725000 | 200 | ENSDARG00000056480 |
| 289 | 16 | 21650000 | 21675000 | 21550000 | 21575000 | 100 | ENSDARG00000061789 |
| 290 | 16 | 23775000 | 23800000 | 23550000 | 23575000 | 225 | ENSDARG00000055475 |
| 291 | 16 | 24500000 | 24525000 | 24300000 | 24325000 | 200 | ENSDARG00000097689 |
| 292 | 16 | 33800000 | 33825000 | 25925000 | 25950000 | 7875 | ENSDARG00000104340 |
| 293 | 16 | 27150000 | 27175000 | 27350000 | 27375000 | 200 | ENSDARG00000108060 |
| 294 | 16 | 30300000 | 30325000 | 29725000 | 29750000 | 575 | ENSDARG00000112961 |
| 295 | 16 | 32310000 | 32320000 | 32560000 | 32570000 | 250 | ENSDARG00000097514 |
| 296 | 16 | 33575000 | 33600000 | 33400000 | 33425000 | 175 | ENSDARG00000092272 |
| 297 | 16 | 36975000 | 37000000 | 37875000 | 37900000 | 900 | ENSDARG00000039989 |
| 298 | 16 | 40325000 | 40350000 | 40075000 | 40100000 | 250 | ENSDARG00000070081 |
| 299 | 16 | 40325000 | 40350000 | 40075000 | 40100000 | 250 | ENSDARG00000070085 |
| 300 | 16 | 40325000 | 40350000 | 40075000 | 40100000 | 250 | ENSDARG00000097565 |
| 301 | 16 | 42470000 | 42480000 | 42780000 | 42790000 | 310 | ENSDARG00000040130 |
| 302 | 16 | 42470000 | 42480000 | 42780000 | 42790000 | 310 | ENSDARG00000103484 |
| 303 | 16 | 42710000 | 42720000 | 42760000 | 42770000 | 50 | ENSDARG00000073952 |
| 304 | 16 | 42870000 | 42875000 | 46100000 | 46105000 | 3230 | ENSDARG00000070000 |
| 305 | 16 | 48700000 | 48725000 | 48400000 | 48425000 | 300 | ENSDARG00000079766 |
| 306 | 16 | 48400000 | 48425000 | 48700000 | 48725000 | 300 | ENSDARG00000102452 |
| 307 | 16 | 52950000 | 52975000 | 53400000 | 53425000 | 450 | ENSDARG00000025043 |
| 308 | 17 | 830000 | 840000 | 660000 | 670000 | 170 | ENSDARG00000114043 |
| 309 | 17 | 825000 | 850000 | 950000 | 975000 | 125 | ENSDARG00000114043 |
| 310 | 17 | 11160000 | 11170000 | 11350000 | 11360000 | 190 | ENSDARG00000097772 |
| 311 | 17 | 14450000 | 14475000 | 14575000 | 14600000 | 125 | ENSDARG00000028586 |
| 312 | 17 | 14700000 | 14725000 | 14450000 | 14475000 | 250 | ENSDARG00000003925 |
| 313 | 17 | 14450000 | 14475000 | 14700000 | 14725000 | 250 | ENSDARG00000028586 |
| 314 | 17 | 14775000 | 14800000 | 15025000 | 15050000 | 250 | ENSDARG00000009253 |
| 315 | 17 | 14775000 | 14800000 | 15025000 | 15050000 | 250 | ENSDARG00000057681 |
| 316 | 17 | 17250000 | 17275000 | 16800000 | 16825000 | 450 | ENSDARG00000043746 |
| 317 | 17 | 26600000 | 26625000 | 26800000 | 26825000 | 200 | ENSDARG00000007285 |
| 318 | 17 | 26800000 | 26825000 | 26600000 | 26625000 | 200 | ENSDARG00000061169 |
| 319 | 17 | 26800000 | 26825000 | 26600000 | 26625000 | 200 | ENSDARG00000097490 |
| 320 | 17 | 26600000 | 26625000 | 26800000 | 26825000 | 200 | ENSDARG00000116527 |
| 321 | 17 | 27270000 | 27280000 | 27330000 | 27340000 | 60 | ENSDARG00000054823 |
| 322 | 17 | 27400000 | 27410000 | 27350000 | 27360000 | 50 | ENSDARG00000107926 |
| 323 | 17 | 29100000 | 29125000 | 28950000 | 28975000 | 150 | ENSDARG00000070769 |
| 324 | 17 | 30410000 | 30420000 | 30490000 | 30500000 | 80 | ENSDARG00000070794 |
| 325 | 17 | 35225000 | 35250000 | 33950000 | 33975000 | 1275 | ENSDARG00000010181 |
| 326 | 17 | 34800000 | 34825000 | 34375000 | 34400000 | 425 | ENSDARG00000097163 |
| 327 | 17 | 44450000 | 44475000 | 44075000 | 44100000 | 375 | ENSDARG00000031533 |
| 328 | 17 | 44450000 | 44475000 | 44550000 | 44575000 | 100 | ENSDARG00000031533 |
| 329 | 18 | 10950000 | 10975000 | 11350000 | 11375000 | 400 | ENSDARG00000042188 |
| 330 | 18 | 14700000 | 14710000 | 14770000 | 14780000 | 70 | ENSDARG00000026664 |
| 331 | 18 | 16125000 | 16150000 | 15900000 | 15925000 | 225 | ENSDARG00000057698 |
| 332 | 18 | 16700000 | 16725000 | 16325000 | 16350000 | 375 | ENSDARG00000057167 |
| 333 | 18 | 16700000 | 16725000 | 16325000 | 16350000 | 375 | ENSDARG00000080895 |
| 334 | 18 | 17775000 | 17800000 | 18050000 | 18075000 | 275 | ENSDARG00000069122 |
| 335 | 18 | 22675000 | 22700000 | 22400000 | 22425000 | 275 | ENSDARG00000056525 |
| 336 | 18 | 22400000 | 22425000 | 22675000 | 22700000 | 275 | ENSDARG00000056628 |
| 337 | 18 | 38750000 | 38760000 | 38380000 | 38390000 | 370 | ENSDARG00000093233 |
| 338 | 18 | 40790000 | 40800000 | 40850000 | 40860000 | 60 | ENSDARG00000017105 |
| 339 | 18 | 49075000 | 49100000 | 48675000 | 48700000 | 400 | ENSDARG00000099137 |
| 340 | 19 | 3850000 | 3875000 | 12050000 | 12075000 | 8200 | ENSDARG00000100698 |
| 341 | 19 | 3930000 | 3940000 | 4010000 | 4020000 | 80 | ENSDARG00000104701 |
| 342 | 19 | 19750000 | 19760000 | 18150000 | 18160000 | 1600 | ENSDARG00000105013 |
| 343 | 19 | 28775000 | 28800000 | 28475000 | 28500000 | 300 | ENSDARG00000004588 |
| 344 | 19 | 28775000 | 28800000 | 28475000 | 28500000 | 300 | ENSDARG00000096389 |
| 345 | 19 | 28775000 | 28800000 | 29250000 | 29275000 | 475 | ENSDARG00000004588 |
| 346 | 19 | 28775000 | 28800000 | 29250000 | 29275000 | 475 | ENSDARG00000096389 |
| 347 | 19 | 29875000 | 29900000 | 30375000 | 30400000 | 500 | ENSDARG00000044980 |
| 348 | 19 | 31000000 | 31025000 | 31325000 | 31350000 | 325 | ENSDARG00000029058 |
| 349 | 19 | 35360000 | 35370000 | 32820000 | 32830000 | 2540 | ENSDARG00000006290 |
| 350 | 19 | 41960000 | 41970000 | 42040000 | 42050000 | 80 | ENSDARG00000019426 |
| 351 | 19 | 47475000 | 47500000 | 47300000 | 47325000 | 175 | ENSDARG00000098992 |
| 352 | 20 | 2950000 | 2975000 | 2750000 | 2775000 | 200 | ENSDARG00000043856 |
| 353 | 20 | 2950000 | 2975000 | 2750000 | 2775000 | 200 | ENSDARG00000043858 |
| 354 | 20 | 23025000 | 23050000 | 12500000 | 12525000 | 10525 | ENSDARG00000115492 |
| 355 | 20 | 16450000 | 16475000 | 16625000 | 16650000 | 175 | ENSDARG00000011000 |
| 356 | 20 | 26550000 | 26575000 | 26675000 | 26700000 | 125 | ENSDARG00000055374 |
| 357 | 20 | 28880000 | 28890000 | 28930000 | 28940000 | 50 | ENSDARG00000027734 |
| 358 | 20 | 28880000 | 28890000 | 28930000 | 28940000 | 50 | ENSDARG00000030116 |
| 359 | 20 | 30375000 | 30400000 | 30550000 | 30575000 | 175 | ENSDARG00000042566 |
| 360 | 20 | 30375000 | 30400000 | 30550000 | 30575000 | 175 | ENSDARG00000080823 |
| 361 | 20 | 30375000 | 30400000 | 30550000 | 30575000 | 175 | ENSDARG00000083108 |
| 362 | 20 | 36775000 | 36800000 | 36675000 | 36700000 | 100 | ENSDARG00000018257 |
| 363 | 20 | 37570000 | 37580000 | 37630000 | 37640000 | 60 | ENSDARG00000096774 |
| 364 | 20 | 38500000 | 38510000 | 38450000 | 38460000 | 50 | ENSDARG00000096730 |
| 365 | 20 | 38900000 | 38910000 | 39010000 | 39020000 | 110 | ENSDARG00000081311 |
| 366 | 20 | 48475000 | 48500000 | 49000000 | 49025000 | 525 | ENSDARG00000091756 |
| 367 | 20 | 52725000 | 52750000 | 52050000 | 52075000 | 675 | ENSDARG00000039652 |
| 368 | 21 | 575000 | 600000 | 700000 | 725000 | 125 | ENSDARG00000100549 |
| 369 | 21 | 1725000 | 1750000 | 2700000 | 2725000 | 975 | ENSDARG00000090387 |
| 370 | 21 | 4200000 | 4225000 | 4100000 | 4125000 | 100 | ENSDARG00000015971 |
| 371 | 21 | 5975000 | 6000000 | 6075000 | 6100000 | 100 | ENSDARG00000044807 |
| 372 | 21 | 20725000 | 20750000 | 12500000 | 12525000 | 8225 | ENSDARG00000079031 |
| 373 | 21 | 17120000 | 17130000 | 17190000 | 17200000 | 70 | ENSDARG00000113703 |
| 374 | 21 | 18025000 | 18050000 | 18250000 | 18275000 | 225 | ENSDARG00000044718 |
| 375 | 21 | 19680000 | 19690000 | 19740000 | 19750000 | 60 | ENSDARG00000092538 |
| 376 | 21 | 20225000 | 20250000 | 20100000 | 20125000 | 125 | ENSDARG00000001906 |
| 377 | 21 | 27350000 | 27375000 | 27000000 | 27025000 | 350 | ENSDARG00000012723 |
| 378 | 21 | 27350000 | 27375000 | 27000000 | 27025000 | 350 | ENSDARG00000020676 |
| 379 | 21 | 29225000 | 29250000 | 29100000 | 29125000 | 125 | ENSDARG00000011317 |
| 380 | 21 | 29100000 | 29125000 | 29225000 | 29250000 | 125 | ENSDARG00000091692 |
| 381 | 21 | 30030000 | 30040000 | 30080000 | 30090000 | 50 | ENSDARG00000075526 |
| 382 | 21 | 30030000 | 30040000 | 30080000 | 30090000 | 50 | ENSDARG00000114922 |
| 383 | 21 | 30100000 | 30125000 | 30250000 | 30275000 | 150 | ENSDARG00000015033 |
| 384 | 21 | 45580000 | 45590000 | 45050000 | 45060000 | 530 | ENSDARG00000113657 |
| 385 | 21 | 45580000 | 45590000 | 45650000 | 45660000 | 70 | ENSDARG00000113657 |
| 386 | 22 | 750000 | 760000 | 330000 | 340000 | 420 | ENSDARG00000013279 |
| 387 | 22 | 1475000 | 1500000 | 2825000 | 2850000 | 1350 | ENSDARG00000098609 |
| 388 | 22 | 1575000 | 1600000 | 2225000 | 2250000 | 650 | ENSDARG00000102099 |
| 389 | 22 | 1900000 | 1925000 | 2525000 | 2550000 | 625 | ENSDARG00000104142 |
| 390 | 22 | 3680000 | 3690000 | 3730000 | 3740000 | 50 | ENSDARG00000097145 |
| 391 | 22 | 4625000 | 4650000 | 4025000 | 4050000 | 600 | ENSDARG00000098237 |
| 392 | 22 | 10770000 | 10780000 | 10840000 | 10850000 | 70 | ENSDARG00000058297 |
| 393 | 22 | 10950000 | 10960000 | 11050000 | 11060000 | 100 | ENSDARG00000071524 |
| 394 | 22 | 16150000 | 16160000 | 16030000 | 16040000 | 120 | ENSDARG00000019998 |
| 395 | 22 | 16030000 | 16040000 | 16150000 | 16160000 | 120 | ENSDARG00000042689 |
| 396 | 22 | 18250000 | 18255000 | 18305000 | 18310000 | 55 | ENSDARG00000083496 |
| 397 | 22 | 18490000 | 18500000 | 18440000 | 18450000 | 50 | ENSDARG00000112679 |
| 398 | 22 | 18745000 | 18750000 | 18565000 | 18570000 | 180 | ENSDARG00000018524 |
| 399 | 22 | 20825000 | 20850000 | 20700000 | 20725000 | 125 | ENSDARG00000061992 |
| 400 | 22 | 22025000 | 22050000 | 22125000 | 22150000 | 100 | ENSDARG00000016364 |
| 401 | 22 | 27100000 | 27125000 | 32025000 | 32050000 | 4925 | ENSDARG00000029011 |
| 402 | 22 | 30250000 | 30275000 | 30350000 | 30375000 | 100 | ENSDARG00000040874 |
| 403 | 22 | 36830000 | 36840000 | 32040000 | 32050000 | 4790 | ENSDARG00000070930 |
| 404 | 22 | 32225000 | 32250000 | 32500000 | 32525000 | 275 | ENSDARG00000063177 |
| 405 | 23 | 6310000 | 6320000 | 6180000 | 6190000 | 130 | ENSDARG00000025206 |
| 406 | 23 | 9900000 | 9925000 | 10600000 | 10625000 | 700 | ENSDARG00000030262 |
| 407 | 23 | 17325000 | 17350000 | 17200000 | 17225000 | 125 | ENSDARG00000037607 |
| 408 | 23 | 17780000 | 17785000 | 17720000 | 17725000 | 60 | ENSDARG00000097211 |
| 409 | 23 | 18550000 | 18575000 | 18400000 | 18425000 | 150 | ENSDARG00000017659 |
| 410 | 23 | 18400000 | 18425000 | 18550000 | 18575000 | 150 | ENSDARG00000019205 |
| 411 | 23 | 27130000 | 27140000 | 27230000 | 27240000 | 100 | ENSDARG00000057062 |
| 412 | 23 | 28170000 | 28180000 | 28290000 | 28300000 | 120 | ENSDARG00000009899 |
| 413 | 23 | 28475000 | 28500000 | 28375000 | 28400000 | 100 | ENSDARG00000088398 |
| 414 | 23 | 36100000 | 36125000 | 36250000 | 36275000 | 150 | ENSDARG00000070343 |
| 415 | 24 | 4430000 | 4440000 | 4000000 | 4010000 | 430 | ENSDARG00000116895 |
| 416 | 24 | 5700000 | 5725000 | 5325000 | 5350000 | 375 | ENSDARG00000055629 |
| 417 | 24 | 7525000 | 7550000 | 7325000 | 7350000 | 200 | ENSDARG00000079312 |
| 418 | 24 | 13250000 | 13275000 | 13025000 | 13050000 | 225 | ENSDARG00000058730 |
| 419 | 24 | 19575000 | 19600000 | 19675000 | 19700000 | 100 | ENSDARG00000038428 |
| 420 | 24 | 20950000 | 20975000 | 21925000 | 21950000 | 975 | ENSDARG00000016360 |
| 421 | 24 | 21925000 | 21950000 | 20950000 | 20975000 | 975 | ENSDARG00000058394 |
| 422 | 24 | 21925000 | 21950000 | 23825000 | 23850000 | 1900 | ENSDARG00000058394 |
| 423 | 24 | 28425000 | 28450000 | 28600000 | 28625000 | 175 | ENSDARG00000071492 |
| 424 | 25 | 2575000 | 2600000 | 10400000 | 10425000 | 7825 | ENSDARG00000096850 |
| 425 | 25 | 6075000 | 6100000 | 5700000 | 5725000 | 375 | ENSDARG00000045932 |
| 426 | 25 | 10625000 | 10650000 | 10775000 | 10800000 | 150 | ENSDARG00000013379 |
| 427 | 25 | 10775000 | 10800000 | 10625000 | 10650000 | 150 | ENSDARG00000089303 |
| 428 | 25 | 16425000 | 16450000 | 16525000 | 16550000 | 100 | ENSDARG00000028159 |
| 429 | 25 | 16525000 | 16550000 | 16425000 | 16450000 | 100 | ENSDARG00000086840 |
| 430 | 25 | 16640000 | 16650000 | 16690000 | 16700000 | 50 | ENSDARG00000070717 |
| 431 | 25 | 16975000 | 17000000 | 17175000 | 17200000 | 200 | ENSDARG00000051748 |
| 432 | 25 | 22625000 | 22650000 | 22375000 | 22400000 | 250 | ENSDARG00000051874 |
| 433 | 25 | 32475000 | 32500000 | 29000000 | 29025000 | 3475 | ENSDARG00000017034 |
| 434 | 25 | 36475000 | 36500000 | 36175000 | 36200000 | 300 | ENSDARG00000031138 |

**Supplementary References**

Baranasic, D., Hörtenhuber, M., Balwierz, P., Zehnder, T., Mukarram, A., Nepal, C., Várnai, C., Hadzhiev, Y., Jimenez-Gonzalez, A., Li, N., et al. (2022). Multiomic atlas with functional stratification and developmental dynamics of zebrafish cis-regulatory elements. Nature Genetics 54: 1037-1050.

Barrientos, N.B., Shoppell, E.A., Boyd, R.J., Culotta, V.C., and McCallion, A.S. (2024). Optimized CRISPR inhibition and activation opens key avenues for systematic biological exploration in zebrafish. bioRxiv, 2024.2009. 2016.613289.

Cai, X., Zhou, Z., Zhu, J., Liao, Q., Zhang, D., Liu, X., Wang, J., Ouyang, G., and Xiao, W. (2020). Zebrafish Hif3α modulates erythropoiesis via regulation of gata1 to facilitate hypoxia tolerance. Development, 147: dev185116.

Dong, X., Li, J., He, L., Gu, C., Jia, W., Yue, Y., Li, J., Zhang, Q., Chu, L., and Zhao, Q. (2017). Zebrafish Znfl1 proteins control the expression of hoxb1b gene in the posterior neuroectoderm by acting upstream of pou5f3 and sall4 genes. Journal of Biological Chemistry 292: 13045-13055.

Franke, M., De la Calle-Mustienes, E., Neto, A., Almuedo-Castillo, M., Irastorza-Azcarate, M., Acemel, R., Tena, J., Santos-Pereira, J., and Gómez-Skarmeta, J. (2021). CTCF knockout in zebrafish induces alterations in regulatory landscapes and developmental gene expression. Nature Communications 12: 5415.

Fukushima, H.S., Takeda, H., and Nakamura, R. (2019). Targeted in vivo epigenome editing of H3K27me3. Epigenetics & chromatin 12: 17.

Hoshijima, K., Jurynec, M.J., Shaw, D.K., Jacobi, A.M., Behlke, M.A., and Grunwald, D.J. (2019). Highly efficient CRISPR-Cas9-based methods for generating deletion mutations and F0 embryos that lack gene function in zebrafish. Developmental cell 51: 645-657.

Hu, P., Zhao, X., Zhang, Q., Li, W., and Zu, Y. (2018). Comparison of various nuclear localization signal-fused Cas9 proteins and Cas9 mRNA for genome editing in Zebrafish. G3: Genes, Genomes, Genetics 8: 823-831.

Jao, L.E., Wente, S.R., and Chen, W. (2013). Efficient multiplex biallelic zebrafish genome editing using a CRISPR nuclease system. Proceedings of the National Academy of Sciences 110: 13904-13909.

Tanaka, S., Yoshioka, S., Nishida, K., Hosokawa, H., Kakizuka, A., and Maegawa, S. (2018). In vivo targeted single-nucleotide editing in zebrafish. Scientific Reports 8: 11423.

Weuring, W.J., Dilevska, I., Hoekman, J., van de Vondervoort, J., Koetsier, M., van’t Slot, R.H., Braun, K.P., and Koeleman, B.P. (2021). CRISPRa-mediated upregulation of scn1laa during early development causes epileptiform activity and dCas9-associated toxicity. The CRISPR Journal 4: 575-582.
